# The Trisubstituted Isoxazole MMV688766 Exerts Broad-Spectrum Activity against Drug-Resistant Fungal Pathogens through Inhibition of Lipid Homeostasis

**DOI:** 10.1128/mbio.02730-22

**Published:** 2022-10-27

**Authors:** Emily Puumala, Olga Zaslaver, Amy Chen, Dustin Duncan, Meea Fogal, Rebecca S. Shapiro, Mohammad T. Mazhab-Jafari, Luke Whitesell, J. Rafael Montenegro-Burke, Nicole Robbins, Leah E. Cowen

**Affiliations:** a Department of Molecular Genetics, University of Toronto, Toronto, Ontario, Canada; b Donnelly Centre for Cellular and Molecular Research, University of Toronto, Toronto, Ontario, Canada; c Department of Medical Biophysics, University of Toronto, Toronto, Ontario, Canada; d Department of Molecular and Cellular Biology, University of Guelph, Guelph, Ontario, Canada; Yonsei University

**Keywords:** *Candida albicans*, *Candida auris*, *HAL9*, *HSP12*, Pathogen Box, experimental evolution, fungal pathogen, lipid homeostasis

## Abstract

*Candida* species are among the most prevalent causes of systemic fungal infection, posing a growing threat to public health. While Candida albicans is the most common etiological agent of systemic candidiasis, the frequency of infections caused by non*-albicans Candida* species is rising. Among these is Candida auris, which has emerged as a particular concern. Since its initial discovery in 2009, it has been identified worldwide and exhibits resistance to all three principal antifungal classes. Here, we endeavored to identify compounds with novel bioactivity against C. auris from the Medicines for Malaria Venture’s Pathogen Box library. Of the five hits identified, the trisubstituted isoxazole MMV688766 emerged as the only compound displaying potent fungicidal activity against C. auris, as well as other evolutionarily divergent fungal pathogens. Chemogenomic profiling, as well as subsequent metabolomic and phenotypic analyses, revealed that MMV688766 disrupts cellular lipid homeostasis, driving a decrease in levels of early sphingolipid intermediates and fatty acids and a concomitant increase in lysophospholipids. Experimental evolution to further probe MMV688766’s mode of action in the model fungus Saccharomyces cerevisiae revealed that loss of function of the transcriptional regulator *HAL9* confers resistance to MMV688766, in part through the upregulation of the lipid-binding chaperone *HSP12*, a response that appears to assist in tolerating MMV688766-induced stress. The novel mode of action we have uncovered for MMV688766 against drug-resistant fungal pathogens highlights the broad utility of targeting lipid homeostasis to disrupt fungal growth and how screening structurally-diverse chemical libraries can provide new insights into resistance-conferring stress responses of fungi.

## INTRODUCTION

Fungi kill an estimated 1.5 million individuals annually, with *Candida* species being one of the four most predominant genera capable of causing life-threatening infections in humans (1). While Candida albicans currently reigns as the most common cause of systemic candidiasis (2), there has recently been an increase in non-*albicans Candida* infections, including those caused by the emerging drug-resistant pathogen Candida auris (3, 4). Since its discovery in 2009, C. auris has been identified across the globe, clustering into four major clades, South Asian (I), East Asian (II), African (III), and South American (IV), each of which is evolutionarily and geographically distinguishable (5–8). C. auris infections are typically hospital acquired, with an average mortality rate of 60% (9–11). Distinguishing features of C. auris that contribute to its significant impact on public health include its propensity to colonize the human skin, exceptional nosocomial persistence, and prevalence of resistance to at least one antifungal class (12). Specifically, 90% of isolates are reported as azole resistant, 10% are echinocandin resistant, and approximately 4% are resistant to all three major antifungal classes (13). In the age of COVID-19, epidemiological links have also been identified between clinical coinfection with C. auris and prolonged hospitalization due to SARS-CoV-2-related critical illness (14–16). Thus, C. auris poses a grave threat to public health that demands efforts to develop new, more effective therapeutics.

In general, antifungal development has been slow due to the eukaryotic nature of fungal cells, challenges associated with compound permeability across the fungal cell wall and membrane, and limited interest from the pharmaceutical industry in developing novel antifungals (17–19). In fact, a new class of antifungal has not entered clinical practice since the mid-2000s (18, 20, 21). Currently, antifungals used to treat systemic infections target the membrane sterol ergosterol (polyenes), ergosterol biosynthesis (azoles), or the biosynthesis of 1,3-β-d-glucan, a key component in the fungal cell wall (echinocandins). However, the emergence of pathogens such as C. auris, as well as the continued evolution of resistance to all three of these antifungal classes among other pathogenic fungal species, highlights the urgent need for the discovery of novel agents and strategies to address the burden of drug-resistant infections. Fortunately, the continued development and expansion of structurally-diverse compound collections coupled with the expanding suite of functional genomic resources in multiple fungal species (22) have enabled the efficient discovery and mechanistic characterization of candidate antifungals (22).

Leveraging existing small-molecule libraries has become a productive method to identify chemical matter with previously unrecognized antifungal or antifungal-potentiating activity (20). Notable examples include the antidepressant sertraline and oestrogen receptor modulator tamoxifen, which both advanced to late-stage clinical trials to treat infections caused by the fungal pathogen Cryptococcus neoformans (23, 24). Other notable compound collections recently evaluated for antifungal activity include the Malaria Box and the Pathogen Box, both assembled by the Medicines for Malaria Venture (MMV) to bring together diverse molecules that are active against malaria and neglected tropical diseases. Studies have found activity for these molecules against diverse fungal pathogens, including C. albicans, C. auris, and C. neoformans (25–28), highlighting these chemical collections as a rich source of compounds with novel antifungal activity. These screens have largely focused on identifying baseline antifungal activity in diverse fungal growth states (e.g., biofilms versus planktonic cells) (25, 29, 30), without characterizing the antifungal modes of action of hit compounds.

Here, we describe the results of a phenotypic screen of the MMV Pathogen Box to identify compounds with previously unknown bioactivity against C. auris. Screening of this resource identified a trisubstituted isoxazole with potent fungicidal activity against multiple *Candida* species. Subsequent characterization of its mode of action provided new insights into the cytoprotective mechanisms fungi use to cope with the disruption of lipid homeostasis.

## RESULTS

### A trisubstituted isoxazole, MMV688766, exerts potent fungicidal activity against C. auris.

**FIG 1:**
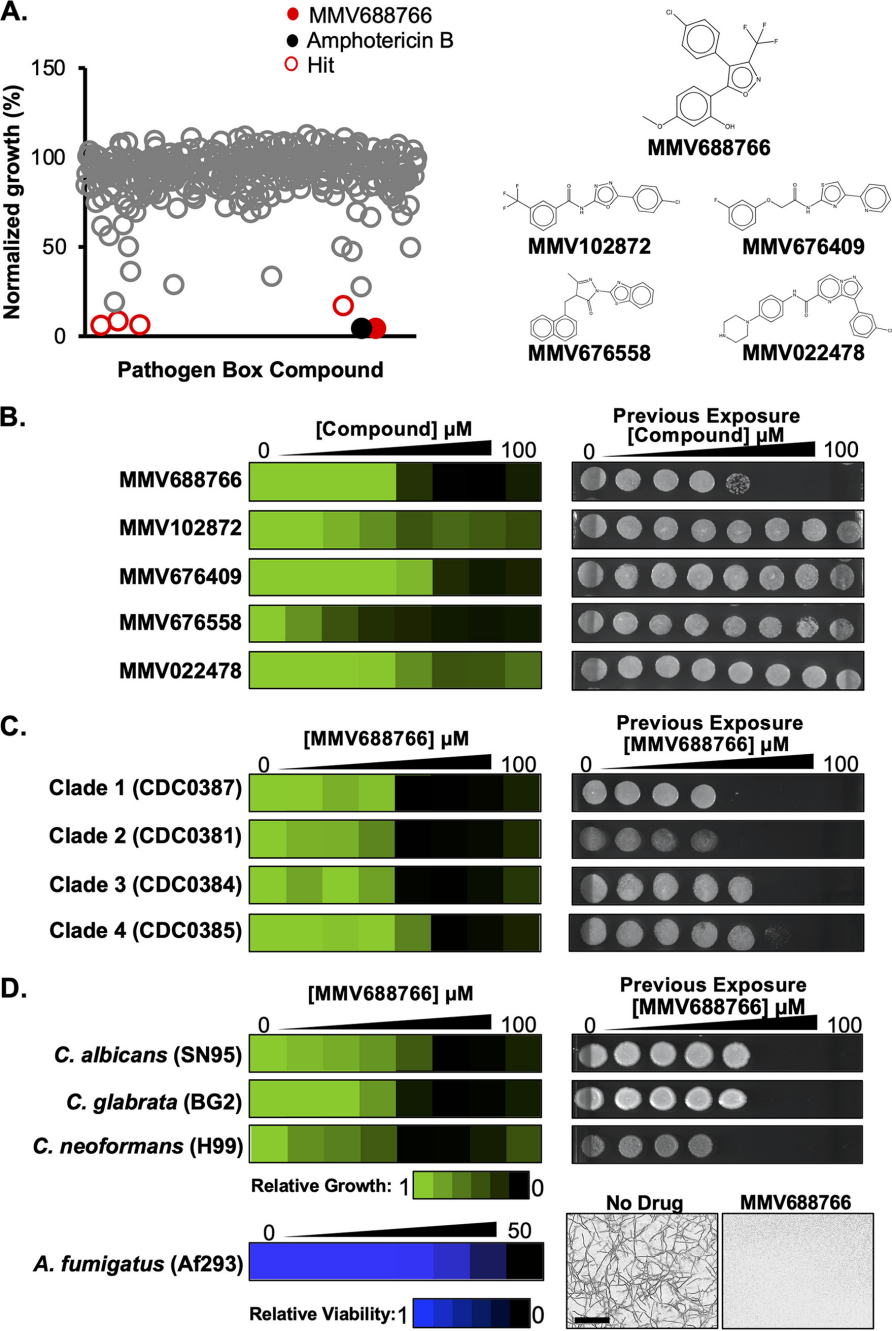
The trisubstituted isoxazole MMV688766 has fungicidal activity against yeasts and molds. (A) Summary of Pathogen Box screen (left). Each of the 400 Pathogen Box compounds was screened at 25 μM in RPMI at 30°C for 48 h against a C. auris clinical isolate (CDC0387) in 96-well plates. Growth was monitored by optical density at 600 nm (OD_600_). Percent growth of treated samples was calculated by normalizing the OD_600_ of treated wells to the average OD_600_ of DMSO solvent controls (16 per plate). Single-agent antifungal hits (red) were identified by a >80% reduction in growth compared to DMSO solvent controls. MMV688766 is indicated in solid red. Amphotericin B control is indicated in black. Structures of each hit compound are shown at right. (B) The five hits were validated for bioactivity using dose-response assays in RPMI at 30°C. Growth was measured using absorbance at 600 nm after 48 h. Technical duplicates were averaged, and measurements were normalized to no-drug controls. Relative growth is quantitatively represented by color (see scale bar at bottom). Fungicidal activity was assessed by removing 3 μL from each well from the dose-response assay after the 48-h incubation, and spotting the inoculum onto drug-free YPD agar. Plates were incubated for 24 h at 30°C. (C) MMV688766 activity was tested against a panel of C. auris isolates from each geographically distinct clade (1 to 4) as described in panel B. (D) MMV688766 activity was tested against C. albicans, C. glabrata, and C. neoformans as described in B. For A. fumigatus, MMV688766 activity was assessed using a 2-fold dose-response assay in RPMI at 37°C for 48 h. Relative viable cell number was quantified by incubating cells with alamarBlue (0.03% wt/vol) after 48 h followed by an additional 24-h incubation at 37°C. Growth of A. fumigatus was also observed microscopically using an IncuCyte live cell imager after 48-h incubation with compound at ×20 magnification (scale bar = 200 μm). All dose-response data are plotted using Java TreeView3; see color bars.

To explore the potential of repurposing existing drug-like molecules for the treatment of C. auris, we performed a small-molecule screen using the Pathogen Box library. Compiled by the MMV as an open-source tool for drug discovery, the Pathogen Box contains 400 drug-like molecules with bioactivity against diverse causative agents of neglected tropical diseases (28, 31). We screened the Pathogen Box at 25 μM in RPMI 1640 medium at 30°C using a clinical isolate of C. auris (CDC0387). Hit molecules were defined by their ability to cause ≥80% growth inhibition of C. auris compared to solvent controls (Fig. 1A). Five structurally-distinct molecules with no previously annotated antifungal activity were identified as single-agent hits from our screen: MMV688766, MMV102872, MMV676409, MMV676558, and MMV022478 (Fig. 1). The bioactivity of each hit was verified through 2-fold dose-response assays to calculate the minimum inhibitory concentration of a compound that resulted in an ≥80% reduction in cell growth (MIC_80_) under similar conditions as the primary screen (Fig. 1B), as well as using Clinical and Laboratory Standards Institute (CLSI) culturing conditions (Fig. S1) (32). We also tested whether the five single-agent hits displayed fungistatic or fungicidal activity, by spotting samples from each dose-response assay well (3 μL) onto drug-free YPD agar after 48 h of compound exposure (Fig. 1B and see Fig. S1A in the supplemental material). YPD agar plates were incubated for an additional 24 h to determine whether viable cells had persisted in the treated inocula. The dose-response assays revealed that MMV688766 and MMV676558 were the most potent molecules against C. auris, inhibiting cell growth by >80% at 25 μM and 12.5 μM, respectively (Fig. 1B and Fig. S1A). However, MMV688766 was the only compound to demonstrate fungicidal activity against C. auris (Fig. 1B and Fig. S1A). Thus, we prioritized this molecule for additional characterization.

10.1128/mbio.02730-22.1 FIG S1 The trisubstituted isoxazole MMV688766 has antifungal activity against C. auris, C. albicans, C. glabrata, and C. neoformans under CLSI standard culture conditions. (A) The five hits from the Pathogen Box screen were validated for bioactivity using dose-response assays in RPMI at 35°C. Growth was measured using absorbance at 600 nm after 48 h. Technical duplicates were averaged, and measurements were normalized to no-drug controls. Relative growth is quantitatively represented by a colour (see scale bar at bottom). Fungicidal activity was assessed by removing 3 μL from each well from the dose-response assay after the 48-hour incubation and spotting the inoculum onto drug-free YPD agar. Plates were incubated for 24 h at 30°C. (B) MMV688766 activity was tested against a panel of C. auris isolates from each geographically distinct clade (1 to 4) as described in panel A. (C) MMV688766 activity was tested against C. albicans, C. glabrata, and C. neoformans as described in panel A. Growth of the C. neoformans dose-response assay was read after 72 h. All dose-response data are plotted using Java TreeView3; see colour bar. Download FIG S1, PDF file, 0.7 MB. Copyright © 2022 Puumala et al. 2022 Puumala et al. https://creativecommons.org/licenses/by/4.0/ This content is distributed under the terms of the Creative Commons Attribution 4.0 International license.

To interrogate its spectrum of activity, dose-response assays were first performed with MMV688766 against the four geographically distinct C. auris clades (Fig. 1C and Fig. S1B). The compound inhibited the growth of multiple isolates spanning all four clades, with MIC_80_ values between 12.5 and 50 μM depending on the temperature of incubation (Fig. 1C and Fig. S1B and S2A), and was consistently fungicidal against clade I and II isolates. The only exception was the C. auris isolate CDC0389, which was resistant to MMV688766 up to 100 μM (Fig. S2A). Notably, the pleiotropic drug resistance ATP-binding cassette (ABC) pump-encoding gene *CDR1* is upregulated ~4-fold in CDC0389 compared to the screening strain CDC0387 (Fig. S2B). Given its potency against C. auris, we next assessed whether this compound was active against other evolutionarily-diverse fungal pathogens. MMV688766 inhibited the growth of C. albicans, Candida glabrata, and C. neoformans with comparable potency to that observed against C. auris (MIC_80_ = 12.5 to 25 μM; Fig. 1D and Fig. S1C). Spotting inocula from these dose-response assays confirmed the compound was fungicidal against these species as well (Fig. 1D and Fig. S1C). We also assessed the bioactivity of MMV688766 against the filamentous mold Aspergillus fumigatus. As measured by standard resazurin dye reduction assay, treatment with 50 μM MMV688766 resulted in complete inhibition of A. fumigatus growth, which was confirmed visually by microscopy (Fig. 1D). Finally, we sought to determine whether MMV688766 demonstrated mammalian cytotoxicity against a human hepatocellular carcinoma cell line, HepG2 (Fig. S2C), as well as cytotoxicity in a nematode infection model (Fig. S2D). While MMV688766 demonstrated comparable cytotoxicity against HepG2 cells and C. elegans as it did against the fungal pathogens tested, we remained interested in exploring the mechanism of action of this molecule. Ultimately, our screen of the Pathogen Box library against C. auris unveiled a compound that exerts potent activity against *Candida*, Cryptococcus, and Aspergillus.

10.1128/mbio.02730-22.2 FIG S2 MMV688766 has fungicidal activity against isolates of all four geographically distinct clades of Candida auris, and is toxic against mammalian cells as well as C. elegans in a nematode model of C. albicans infection. (A) The potency of MMV688766 was tested against a panel of C. auris isolates from each geographically distinct clade (1 to 4), using a 2-fold dose-response assay in RPMI at 30°C for 48 h. Fungicidal activity was assessed by removing 3 μL from each well from the dose-response assay after the 48-h incubation and spotting the inoculum onto drug-free YPD agar. Plates were incubated for 24 h at 30°C. All dose-response data are displayed using Java TreeView3; see color bars. (B) Transcript levels of *CDR1* in C. auris CDC0387 and CDC0389 was measured by RT-qPCR and normalized to *ACT1* and *GPD1.* Values are relative to the CDC0387 screening strain. Error bars represent standard error of the mean (SEM) among technical triplicates. Significance was measured using a two-tailed unpaired *t* test with Welch’s correction: *, *P < *0.05. (C) Human hepatocellular carcinoma (HepG2) cells were seeded at 5 × 10^4^ cells/mL in RPMI-1640 supplemented with 10% heat-inactivated fetal bovine serum for 24 h at 37°C with 5% CO_2_. A 2-fold dilution series of each compound was added to cells which were treated for 72 h at 37°C with 5% CO_2_. The metabolic dye alamarBlue was added after treatment for 3 h at 37°C, and the fluorescent signal was measured at excitation/emission 560 nm/590 nm and corrected for background fluorescence from the medium. Percent cell viability in the presence of each compound was normalized to drug-free controls and plotted (red) alongside DMSO (blue, negative) and topotecan (gray, positive) controls. Error bars represent standard deviation of technical triplicates (*n *=* *3). Similar results were obtained from a biological replicate. (D) *glp-4* nematodes feeding on E. coli (OP05) were washed, counted, and transferred to brain heart infusion (BHI) infection plates with or without C. albicans (SC5314) fungal lawns to feed for 3 h. Infected worms were washed and counted, and 25 worms were added to wells of a 48-well plate and allowed to grow in the presence or absence of 50 μM MMV688766 in 80% M9 buffer, 20% BHI, 10 μg/mL cholesterol, and 90 μg/mL kanamycin. Plates were incubated at 25°C and worm survival was scored at 24 h postinfection (hpi) under a stereomicroscope at ×10 magnification (*n *=* *3; uninfected-untreated *n *=* *1). Download FIG S2, PDF file, 0.7 MB. Copyright © 2022 Puumala et al. 2022 Puumala et al. https://creativecommons.org/licenses/by/4.0/ This content is distributed under the terms of the Creative Commons Attribution 4.0 International license.

### Chemical-genetic profiling suggests MMV688766 targets lipid homeostasis.

**FIG 2:**
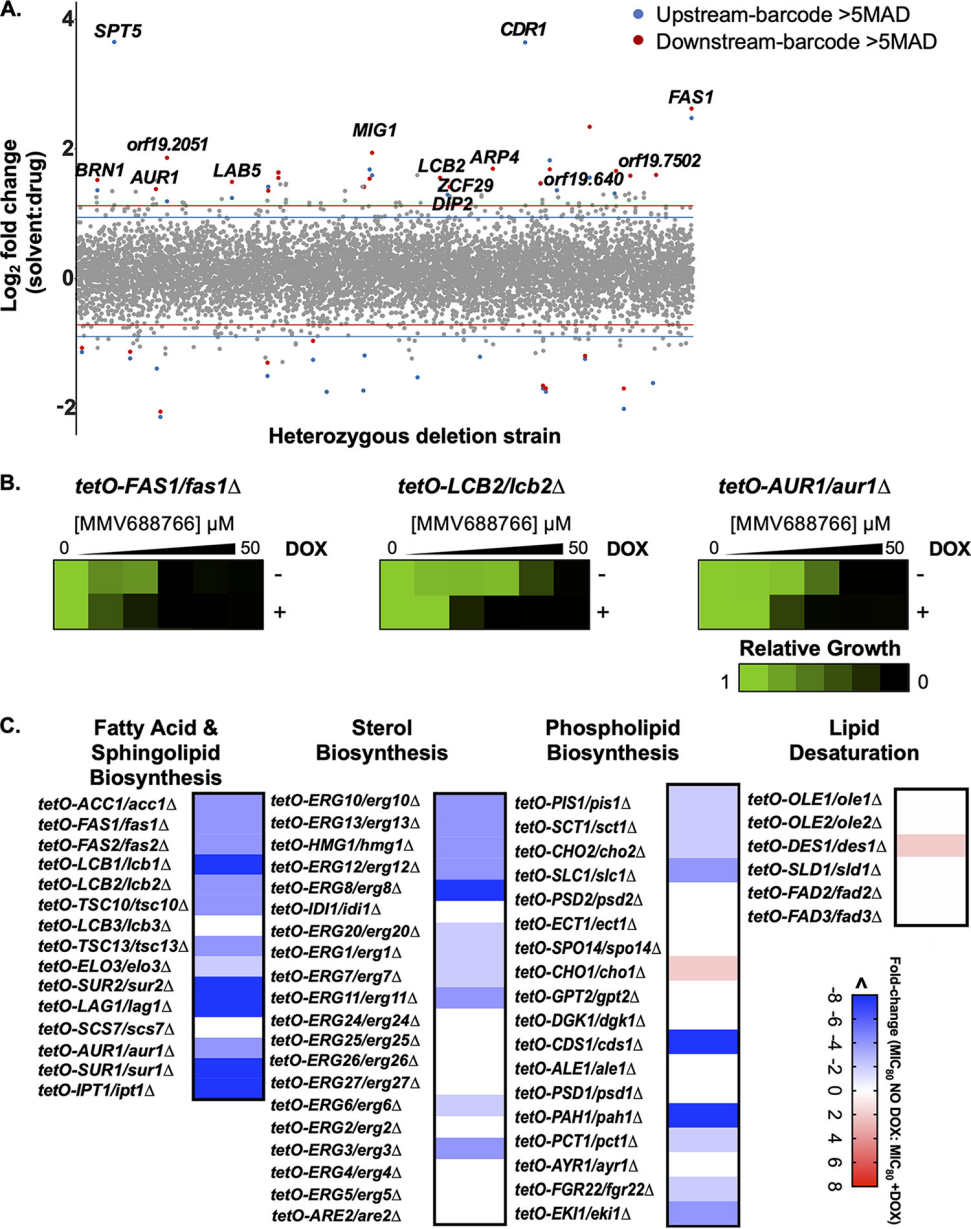
Chemical-genetic profiling in C. albicans suggests MMV688766 impairs lipid homeostasis. (A) A pooled library of C. albicans double-barcoded heterozygous deletion mutants covering ~90% of the C. albicans genome was grown in the presence of 8 μM MMV688766 (IC_20_) or an equivalent volume of the DMSO solvent control. Genomic DNA was isolated and the up- and downstream strain-specific barcodes were amplified and pooled for high-throughput sequencing to evaluate relative abundance of each mutant strain. Strains were considered significantly reduced in abundance if the solvent/drug log_2_ ratio was greater than 5 median absolute deviations (MADs) above the median for both the upstream and the downstream barcodes or if either the upstream or downstream was >5 MADs and the opposing barcode was omitted due to low total reads in the solvent samples. Barcodes significantly altered in abundance are shown in blue (upstream barcode) and red (downstream barcode), and gray represents barcodes that were not significantly altered in abundance. (B) Genetic depletion of lipid biosynthesis genes confers sensitivity to MMV688766. Dose-response assays with 2-fold dilutions of MMV688766 were set up using doxycycline-repressible (*tetO*) mutants in the presence and absence of doxycycline (DOX; 0.05 μg/mL) to facilitate transcriptional repression. After incubation in YPD for 48 h at 30°C, growth was measured by OD_600_ and normalized to drug-free controls. Data were plotted using Java TreeView3; see color bar. (C) Dose-response assays were performed as in panel B using an expanded set of *tetO* mutants, except for essential genes 0.05 μg/mL DOX was used to achieve transcriptional repression and for nonessential genes 20 μg/mL DOX was used. Changes in growth inhibition by MMV688766 in the absence versus presence of DOX were computed as a relative fold change for each mutant and plotted using GraphPad Prism 9; see color bar.

MMV688766 was included in the Pathogen Box due to its bioactivity against the waterborne human intestinal parasite Schistosoma mansoni (31); however, its mechanism of action remains elusive. Fortunately, the potency of MMV688766 against C. albicans allowed us to perform haploinsufficiency profiling (HIP) to explore the mechanism through which this compound exerts its antifungal activity. Target elucidation using HIP operates under the principle that reducing the copy number of the gene encoding a compound’s target, or a gene involved in a pathway related to the compound’s target, will result in hypersensitivity to the compound (33). Triplicate pools of C. albicans double-barcoded heterozygous deletion mutants were grown in the presence of MMV688766 at a concentration that inhibited the growth of the pool by ~20% (8 μM), based on endpoint optical density at 600 nm (OD_600_), compared to solvent-treated pools. High-throughput sequencing of PCR-amplified barcodes was performed to assess the relative abundance of each barcoded strain, and strains carrying barcodes with a solvent/drug log_2_ ratio greater than 5 median absolute deviations (MAD) above the median of the total pool were classified as significantly hypersensitive. This analysis identified 24 genes for which reduced genetic dosage conferred hypersensitivity to MMV688766 (Fig. 2A).

The hypersensitivity of each heterozygous deletion mutant identified by HIP was verified by growth curve analysis. Growth curves were performed in the presence and absence of 8 μM MMV688766, confirming the hypersensitivity of 14 heterozygous deletion mutants compared to the drug-treated wild-type control (Fig. S3). Gene ontology (GO) functional enrichment using the *Candida* Genome Database revealed that functions central to lipid synthesis and homeostasis were significantly enriched: palmitoyltransferase activity (background frequency, 0.2%; cluster frequency, 14.3%; *P* value, 0.011; and false discovery rate, 6%) and acyltransferase activity (background frequency, 2.2%; cluster frequency, 21.4%; *P* value, 0.087; and false discovery rate, 20.67%). These functions encompassed hits *FAS1* and *LCB2*, *FAS1*, *LCB2*, and *ARP4*, respectively. Furthermore, based on both the HIP assay and growth curve validations, genes involved in lipid biosynthesis were among the most abundant (Fig. S3). *FAS1*, *LCB2*, *AUR1*, and *LAB5* are all involved in C. albicans lipid biosynthesis with the first three genes being essential for viability. Based on these findings, we hypothesized that MMV688766 may impair fungal lipid homeostasis.

10.1128/mbio.02730-22.3 FIG S3 Growth curve validation of haploinsufficiency profiling for MMV688766. Growth curves were performed in YPD for 24 h at 30°C under static conditions. C. albicans heterozygous deletion mutants in the presence and absence of 8 μM MMV688766 were compared to their parental control strain (CaSS1) in the presence of drug. Results of growth curve analysis were plotted as the ratio of area under the curve (AUC) of each drug-treated mutant strain to the AUC of the corresponding drug-free control, normalized to the parental strain AUC ratio using GraphPad Prism 9. Error bars represent standard deviation of the mean (*n = *4). Significance was measured using a two-tailed unpaired *t* test with Welch’s correction: *, *P < *0.05; **, *P < *0.005; ***, *P < *0.001. Gene Ontology (GO) slim terms were identified using the GO slim process finder on the *Candida* Genome Database. All 14 validated genes fit under the four processes demonstrated above, and the lipid biosynthetic process (outlined, black) was of particular interest. Download FIG S3, PDF file, 0.7 MB. Copyright © 2022 Puumala et al. 2022 Puumala et al. https://creativecommons.org/licenses/by/4.0/ This content is distributed under the terms of the Creative Commons Attribution 4.0 International license.

To gain a more thorough understanding of MMV688766’s impact on lipid homeostasis, we leveraged strains from the C. albicans gene replacement and conditional expression (GRACE) collection (34). The collection is composed of mutants where one allele of each target gene is deleted and the remaining allele is under the control of a tetracycline-repressible promoter (*tetO*) (34). Based on strain availability, we confirmed that transcriptional repression of *FAS1*, *LCB2*, and *AUR1* using the tetracycline analog doxycycline (DOX) conferred hypersensitivity to MMV688766, consistent with what had been observed with the heterozygous deletion mutants (Fig. 2B). We next expanded this analysis to those genes present in the GRACE collection that are involved in diverse aspects of lipid biosynthesis, including fatty acid, sphingolipid, sterol, and phospholipid biosynthesis. The lowest concentration of MMV688766 that inhibited growth by 80% (MIC_80_) was compared in the DOX-treated versus untreated condition and plotted based on fold change in MIC_80_ (Fig. 2C). Interestingly, we observed that transcriptional repression of multiple genes in the fatty acid and sphingolipid biosynthesis pathways, as well as the early ergosterol biosynthesis pathway (Fig. 2C), conferred a greater than 4-fold increase in hypersensitivity to MMV688766. Depletion of several genes involved in phospholipid biosynthesis also caused enhanced sensitivity to the compound, particularly *CDS1* and *PAH1*. Overall, our chemical-genetic analyses indicated that disrupting lipid homeostasis in C. albicans results in enhanced susceptibility to MMV688766.

### MMV688766 disrupts fatty acid homeostasis without directly inhibiting the canonical fatty acid synthase.

**FIG 3:**
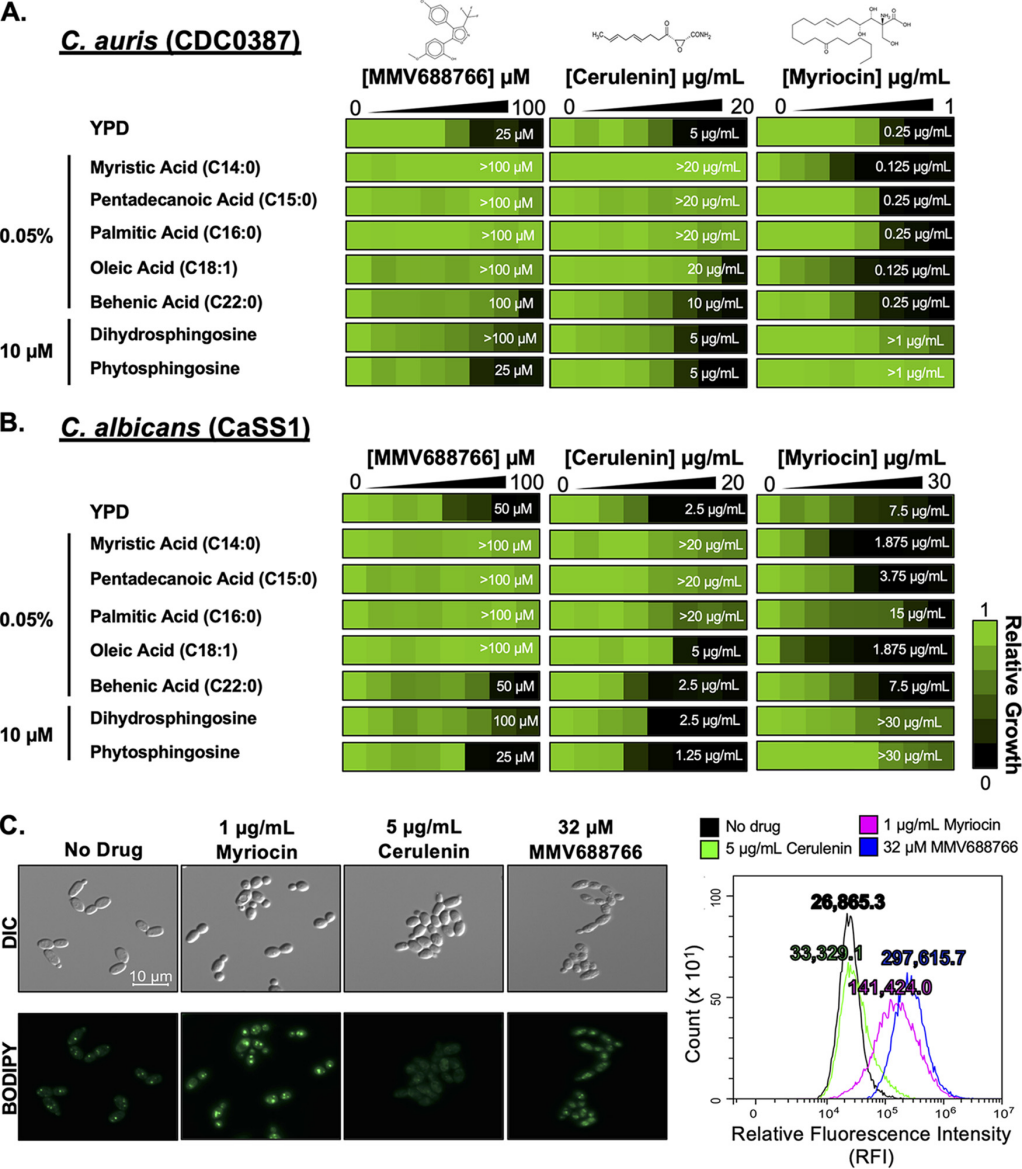
Supplementation with exogenous fatty acids rescues MMV688766-mediated growth inhibition in *Candida*. Two-fold dilutions of MMV688766 (0 to 100 μM), the fatty acid synthase inhibitor cerulenin (0 to 20 μg/mL), and the sphingolipid biosynthesis inhibitor myriocin (0 to 1 μg/mL) were performed against C. auris (CDC0387) (A) and C. albicans (CaSS1) (B) in YPD medium, in the presence and absence of exogenous fatty acids of various chain length (myristic acid (C14:0), pentadecanoic acid (C15:0), palmitic acid (C16:0), oleic acid (C18:1), and behenic acid (C22:0)), as well as the early sphingolipid intermediates, dihydrosphingosine, and phytosphingosine. Growth was measured as OD_600_ following a 48-h incubation at 30°C and normalized to the growth of drug-free control wells. Data are displayed using Java TreeView3; see color bar. MIC_80_ values for each experimental condition are indicated by white numbers on the heat map. (C) C. auris (CDC0387) was grown in the presence and absence of 32 μM MMV688766, 1 μg/mL myriocin, and 5 μg/mL cerulenin for 4 h at 30°C in YPD following a 2-h outgrowth. Cells were washed and stained with 1 μg/mL BODIPY 493/503 to visualize neutral lipid droplets using differential interference contrast (DIC) and an EGFP fluorescence filter to visualize BODIPY staining using a Zeiss Axio Imager.MI at ×100 magnification. Flow cytometry was performed to quantify relative neutral lipid levels in cells exposed to each treatment. Cells were analyzed for incorporation of BODIPY 493/503 measuring relative fluorescence intensity (RFI) and intact cells were gated away from debris. Distributions of fluorescence intensity across each population are plotted in the histogram (no drug: black, cerulenin: green, myriocin: magenta, 32 μg/mL MMV688766: blue). Values describing the mean RFI across each treatment, are included above each peak.

If the growth inhibitory effects observed upon MMV688766 treatment were caused by the depletion of key lipids important for normal cellular function, supplementation with exogenous lipids should mitigate the effect of MMV688766 treatment. To test this hypothesis, we performed dose-response assays in both C. auris (Fig. 3A) and C. albicans (Fig. 3B) using the fatty acid biosynthesis inhibitor cerulenin, the sphingolipid biosynthesis inhibitor myriocin, and MMV688766 in the absence and presence of fatty acids ranging from 14 to 22 carbons, as well as the early sphingolipid intermediates dihydrosphingosine (DHS) and phytosphingosine (PHS). These lipids were selected for supplementation studies because of transcriptional repression of several C. albicans genes involved in fatty acid and sphingolipid biosynthesis increased susceptibility to MMV688766 (Fig. 2B and C). As anticipated, the inhibitory effects of cerulenin were most effectively abrogated upon the addition of the saturated fatty acids myristic (C14:0), pentadecanoic (C15:0), and palmitic acid (C16:0) in both species (Fig. 3A and B) (35). The very long-chain fatty acid behenic acid (C22:0) as well as sphingosine precursors PHS and DHS, did not affect the potency of cerulenin (Fig. 3A and B) (35, 36). In contrast, the inhibitory effects of myriocin were only reduced upon supplementation with sphingosine precursors in C. auris and C. albicans (Fig. 3A and B). Interestingly, the potency of MMV688766 was reduced in the presence of all fatty acids as well as in the presence of DHS in both *Candida* species (Fig. 3A and B), suggesting a mechanism of action distinct from that of the control compounds.

Lipid droplets are dynamic storage organelles that support homeostasis by sequestering excess free lipid species, thus providing protection from lipotoxicity. Based on our observations that MMV688766 alters lipid homeostasis in the cell, we investigated whether MMV688766 treatment caused an accumulation of lipid droplets in C. auris and C. albicans. We assessed lipid droplet accumulation by staining cells with BODIPY 493/503, a marker for neutral lipid droplet accumulation, in the absence and presence of the fatty acid synthase inhibitor cerulenin, the sphingolipid biosynthesis inhibitor myriocin, and MMV688766 (Fig. 3C). Visualization by fluorescence microscopy and quantification by flow cytometry both demonstrated that MMV688766 induced lipid droplet formation in C. auris, in a manner similar to the sphingolipid biosynthesis inhibitor myriocin (Fig. 3C) (37). Further investigation indicated MMV688766 treatment induces lipid droplet accumulation in both C. auris and C. albicans in a concentration-dependent manner (Fig. S4A and B). Treatment with cerulenin did not induce lipid droplet formation in either species, (Fig. S4A and B). Collectively, these data support a model in which MMV688766 alters lipid homeostasis in *Candida* species in a manner that is distinct from that of myriocin and cerulenin.

10.1128/mbio.02730-22.4 FIG S4 MMV688766 induces accumulation of lipid droplets in C. auris and C. albicans in a dose-dependent manner, while loss of function of *HAL9* results in reduction of lipid droplets in the presence of MMV688766 and does not enhance Nile Red efflux. Staining for accumulation of lipid droplets was performed in C. auris (CDC0387) (A) and C. albicans (CaSS1) (B). Cultures were grown in the presence and absence of three concentrations of MMV688766 (16 μM, 24 μM, 32 μM), as well as the known sphingolipid biosynthesis inhibitor myriocin (C. auris: 1 μg/mL; C. albicans: 5 μg/mL), and fatty acid biosynthesis inhibitor cerulenin (5 μg/mL), for 4 h at 30°C in YPD following a 2-h outgrowth. Cells were washed and stained with 1 μg/mL BODIPY 493/503 to visualize neutral lipid droplets using differential interference contrast (DIC) and an EGFP fluorescence filter to visualize BODIPY staining using a Zeiss Axio Imager.MI at 100× magnification. (C) Cultures of 16ABC and (D) *HAL9*^A1543T^ S. cerevisiae strains were grown in the presence and absence of two concentrations of MMV688766 (MMV; 16 μM, 32 μM) for 4 h at 30°C in YPD following a two-hour outgrowth. Cells were washed and stained with 1 μg/mL BODIPY 493/503 to visualize neutral lipid droplets using differential interference contrast (DIC) and an EGFP fluorescence filter using a Zeiss Axio Imager.MI at 100× magnification. (E) Efflux was evaluated through accumulation of the dye, Nile Red, using wild-type S. cerevisiae (BY4741), the 16ABC evolution parent, and three resistant lineages with mutations in *HAL9*. Cells were treated with Nile Red and assessed through flow cytometry. Histograms depict the relative fluorescence intensity (RFI; PE-A) of events. Table summarizes the number of events measured, the mean RFI of each sample (Median Nile Red Accumulation PE-A), and the fold change in mean RFI of strains relative to the wild-type, efflux-competent BY4741 S. cerevisiae strain. Download FIG S4, PDF file, 0.7 MB. Copyright © 2022 Puumala et al. 2022 Puumala et al. https://creativecommons.org/licenses/by/4.0/ This content is distributed under the terms of the Creative Commons Attribution 4.0 International license.

**FIG 4:**
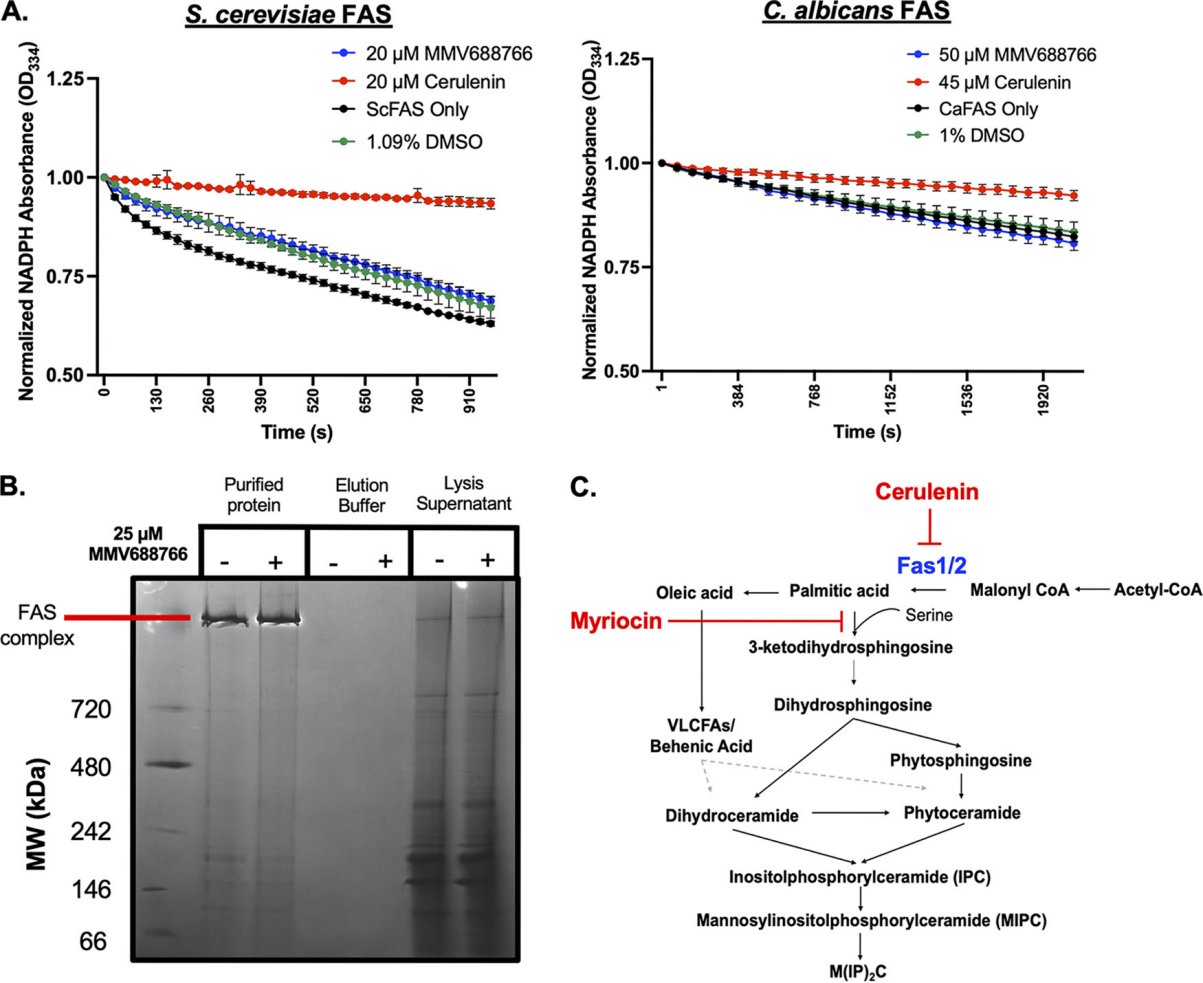
MMV688766 does not directly inhibit fungal fatty acid synthase. (A) S. cerevisiae FAS complex was purified from a 3xFLAG-tagged Fas1 (JWL01) strain (right). C. albicans FAS complex purified from a 6His-3xFLAG-tagged Fas1 strain (CaLC5425) (left). Activity was assayed for activity through spectrophotometric measurement of NADPH oxidation (334 nm) in the absence (black) and presence of 20 μM (*Sc*FAS) or 50 μM (*Ca*FAS) MMV688766 (blue), positive-control compound 20 μM (*Sc*FAS) or 45 μM (*Ca*FAS) cerulenin (red), and DMSO solvent control (green). The mean of triplicate samples is presented. Error bars depict standard deviation and data are plotted using GraphPad Prism 9. (B) C. albicans FAS purified from a 6His-3xFLAG-tagged Fas1 strain (CaLC5425) grown in the presence and absence of 25 μM MMV688766 was run on a 3 to 12% blue Native PAGE gel to determine the integrity of the complex upon treatment. (C) Simplified fungal sphingolipid biosynthetic pathway demonstrating the inhibition modes of cerulenin and myriocin. The pathway was adapted and modified from reference 37.

Based on evidence from HIP, and the strong rescue of C. auris and C. albicans growth conferred by exogenous fatty acids in the presence of MMV688766, we next determined whether MMV688766 directly inhibits fungal fatty acid synthase (FAS). In yeast, the FAS enzyme is made up of α (Fas2) and β (Fas1) subunits organized into a hexameric *α6β6* complex, which synthesizes saturated fatty acids with acyl-chains of up to 18 carbons long from acetyl-CoA and malonyl-CoA in the presence of NADPH (Fig. 4C) (38–40). We assessed whether MMV688766 acted as a direct inhibitor of fungal FAS using FAS complex affinity-purified from a functionally validated S. cerevisiae *FAS1*-3xFLAG strain and a functionally validated *FAS1*-3xFLAG/*FAS1* C. albicans strain (Fig. 4A and B) (41). As a readout for the reductive activity of the affinity-purified complex, NADPH consumption was monitored spectrophotometrically over time in the presence of malonyl-CoA. NADPH is oxidized to NADP^+^ by the enoyl and ketoacyl reductase domains of fungal FAS. As NADPH absorbs light at ~340 nm while NADP^+^ does not, decreased absorbance at 340 nm over time is used as a readout of enzyme activity (39, 41). While cerulenin effectively inhibited *Sc*FAS and *Ca*FAS activity, an equivalent growth-inhibitory concentration of MMV688766 did not inhibit NADPH oxidation relative to the DMSO solvent control in either species (Fig. 4A and B). We also observed that MMV688766 treatment of cells did not cause degradation/destabilization of the FAS complex. Following purification of the C. albicans FAS complex in the presence or absence of 25 μM (>MIC_80_) MMV688766, no alteration in the migration position or intensity of the FAS-containing band visualized by blue Native-PAGE was observed (Fig. 4B). Overall, these data collectively suggest that MMV688766 modulates fatty acid levels without directly inhibiting FAS.

### MMV688766 leads to accumulation of lipid intermediates.

**FIG 5:**
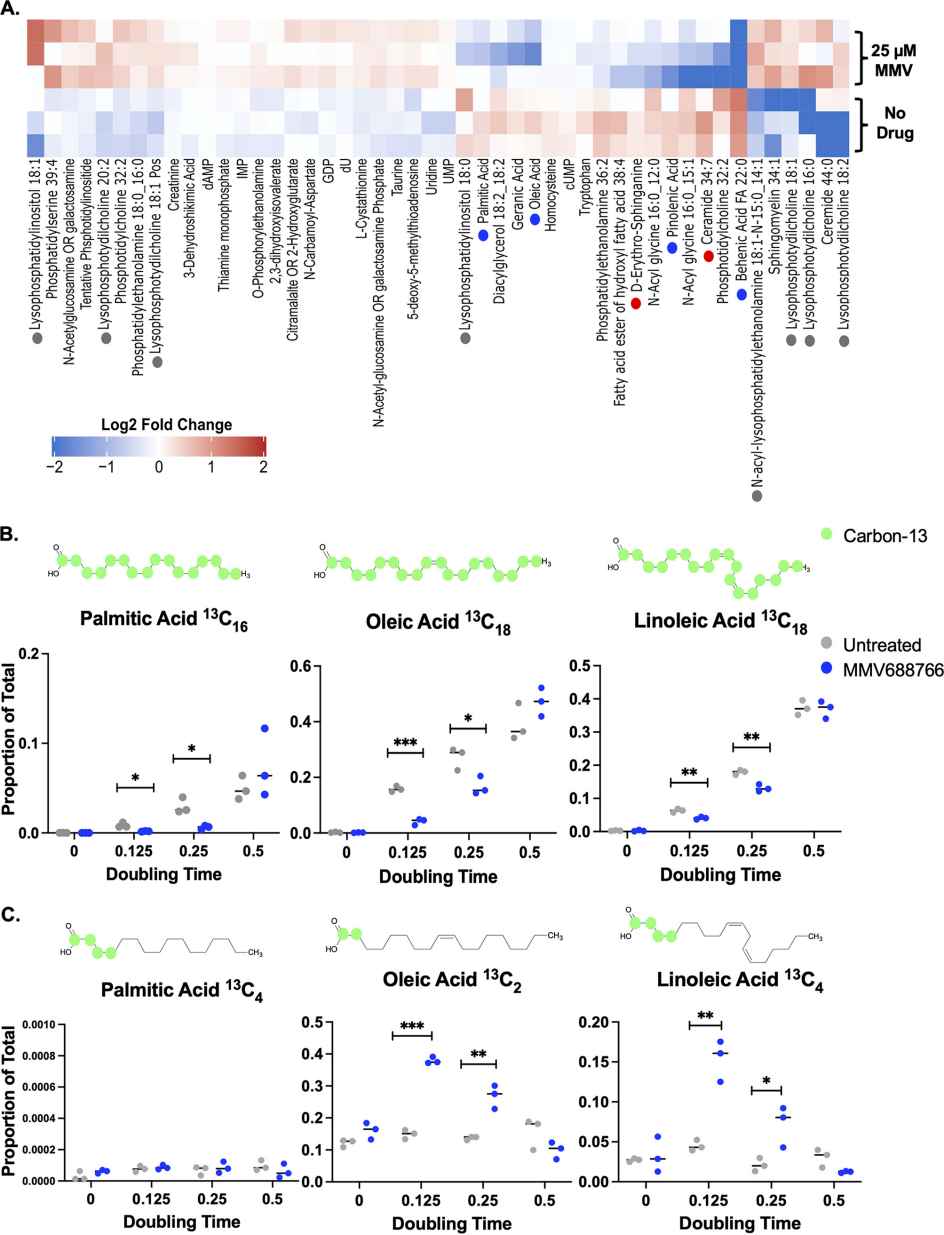
Treatment of C. auris with MMV688766 leads to a reduction in fatty acid and sphingolipid intermediate levels as a result of delayed fatty acids synthesis. (A) C. auris (CDC0387) overnight cultures (*n *=* *3) were diluted to an OD_600_ of ~0.1 and left to grow for 2 h at 30°C in YNB medium supplemented with 2% glucose. Cells were then grown in the presence or absence of 25 μM MMV688766 for an additional 4 h. Lipids were extracted from ~5× 10^8^ cells for each sample, and lipidomics were performed via reverse-phase chromatography for all nonpolar lipids and ion-paired reverse-phase chromatography for all polar lipids. Metabolic features differentially enriched across treatments were selected, and matched using exact mass and MS2 spectral databases using Metaboscape (Bruker). Metabolite levels were median centered and log_2_ transformed (red: increased abundance, blue: decreased abundance see color bar). Blue dots are used to denote fatty acids, gray dots represent lyso-phospholipids, and red dots represent sphingolipid precursors. Samples are plotted in technical triplicate (*n *=* *3). (B) C. auris (CDC0387) samples unlabeled (*t *=* *0) and labeled with ^13^C_2-_acetate grown at 0.125 (*t *=* *22 min), 0.25 (*t *=* *44 min), and 0.5 (*t *=* *88 min) doubling times were evaluated for the ^13^C occupancy of diverse fatty acids using mass spectrometry. The proportion of fully labeled (C-13 depicted as green circles on representative chemical structures) palmitic (^13^C_16_), oleic (^13^C_18_), and linoleic acid (^13^C_18_) compared to total cellular lipids profiled is plotted at each doubling time (blue = 25 μM MMV688766, gray = no drug). (C) The proportion of partially labeled, elongated (C-13 depicted as green circles on representative chemical structures) palmitic (^13^C_4_), oleic (^13^C_2_), and linoleic acid (^13^C_4_) compared to total cellular lipids profiled is plotted at each doubling time (blue = 25 μM MMV688766, gray = no drug). Samples are plotted in biological triplicate (*n *=* *3) and analyzed using unpaired *t test* with Welch’s correction: *, *P < *0.05; **, *P < *0.005; ***, *P < *0.001.

Next, we turned to a metabolomic approach to assess how treatment with MMV688766 modulates cellular lipid levels. Reversed-phase and ion-paired reversed-phase chromatography was used to measure lipid intermediate levels across triplicate MMV688766-treated and untreated samples of C. auris (Fig. 5A). Differentially accumulating metabolic features across the treated and untreated populations were identified by exact mass and MS2 spectral matching against spectral databases using Metaboscape (Bruker), revealing several striking patterns. First, levels of an early biosynthetic sphingolipid intermediate d-erythro-sphinganine, as well as ceramide, were significantly depleted in drug-treated samples (Fig. 5A, red dot). Second, reductions in fatty acid levels were observed in MMV688766-treated samples, including palmitic acid (C16:0), oleic acid (C18:1), and behenic acid (C22:0), all of which feed into the biosynthesis of more complex lipid classes (Fig. 5A, blue dot). Consistent with this observation, MMV688766-treated samples demonstrated an increased abundance of lysophospholipids, which are glycerol-based lipids containing only one fatty acyl moiety at the *sn1* or *sn2* position and a phosphate group at the *sn3* position (Fig. 5A, gray dot) (42). These findings led us to hypothesize that MMV688766 treatment causes depletion of cellular fatty acids, which in turn prevents C. auris from synthesizing the complex lipid classes necessary for maintaining membrane integrity.

To investigate further, we evaluated fatty acid metabolite flux by performing time course labeling experiments using a stable isotope acetate tracer, ^13^C_2_-acetate, and mass spectrometry (Fig. 5B). The relative level of *de novo* synthesis for specific fatty acid species was monitored by detecting ^13^C at each position of the acyl chain. In contrast, partial ^13^C labeling of fatty acid species was considered evidence that elongation of shorter-chain fatty acid precursors had occurred. Flux assays determined that upon culturing C. auris with ^13^C_2_-acetate in the presence of MMV688766, *de novo* fatty acid biosynthesis was delayed, as evidenced by a reduction in the levels of newly synthesized (^13^C-labeled) palmitic acid (C16:0), oleic acid (C18:1) (43), and linoleic acid (C18:2; Fig. 5B). Conversely, we observed that species of oleic and linoleic acids with incomplete ^13^C labeling were significantly higher in abundance in MMV688766-treated cells, compared to the untreated control samples (Fig. 5C). These findings suggest a potential compensatory response to reductions in *de novo* lipid biosynthesis, whereby cells are breaking down existing lipid stores to fatty acyl intermediates and elongating them to produce functional long-chain fatty acid species. It also suggests that during MMV688766 treatment, the fatty acid elongation machinery remains functional in C. auris. The enrichment of lysophospholipid species in our initial steady-state lipidomic profile of samples from drug-treated cells supports this notion (Fig. 5A). Overall, the findings indicate that MMV688766 disrupts fungal fatty acid homeostasis but without direct biochemical inhibition of the FAS complex.

### Loss of function of the zinc-cluster transcription factor *HAL9* confers resistance to MMV688766.

**FIG 6:**
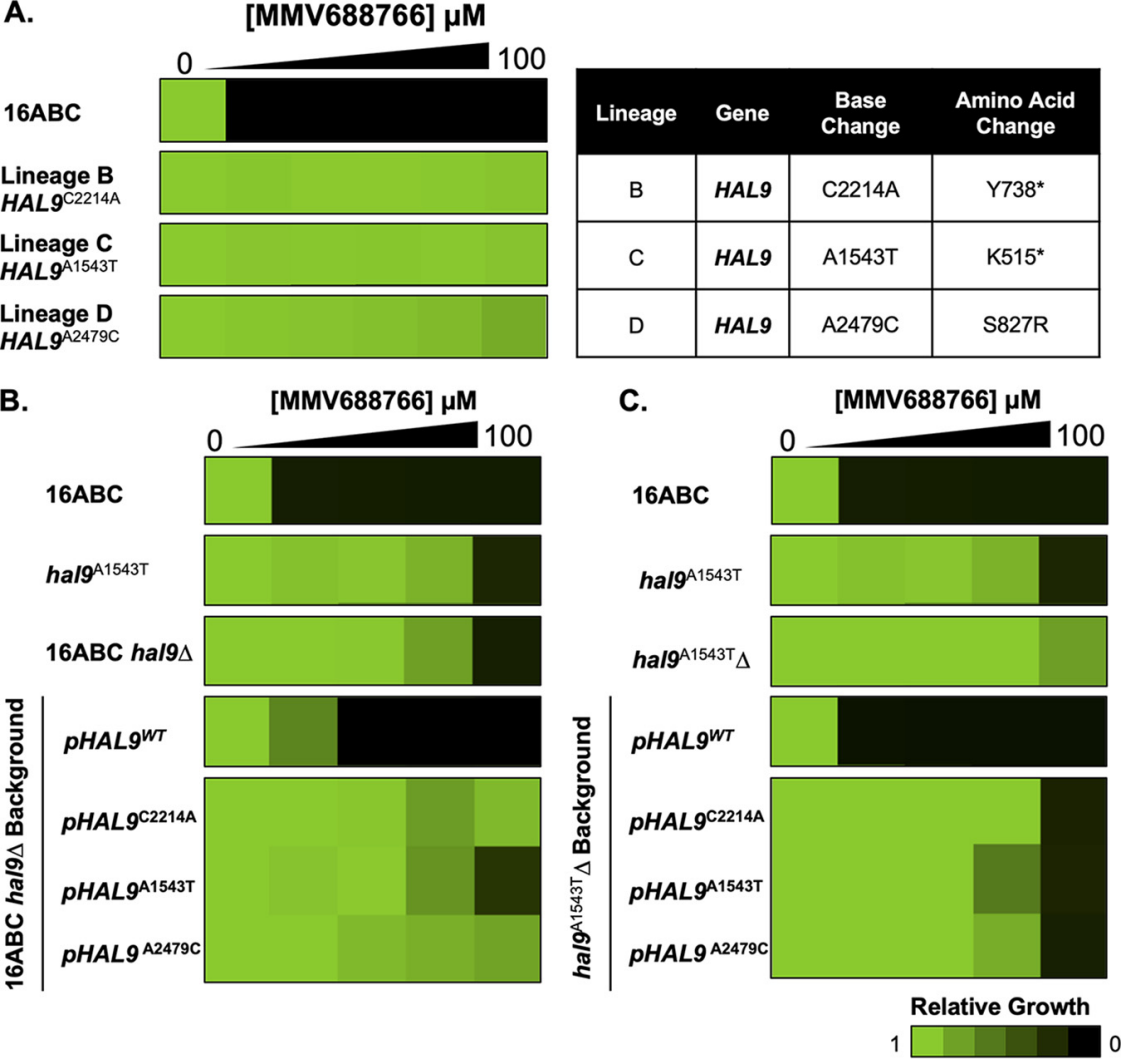
S. cerevisiae MMV688766-resistant lineages carry loss-of-function mutations in the transcription factor *HAL9*. (A) The S. cerevisiae 16ABC strain was used to select for MMV688766-resistant mutants, identified as lineage B, C, and D. The susceptibility of each strain was assessed using a 2-fold dose-response assay under shaking conditions at 30°C in YPD medium. Growth was evaluated by OD_600_ after a 72-h incubation. Mutations in *HAL9* identified by genome sequencing are indicated as nucleotide and amino acid changes. (B) Functional validation of *HAL9* mutations in 16ABC strain background. Assays were performed on allele-swapped mutants as described in A, using synthetic defined medium (SD) to maintain selection of plasmids encoding *HAL9*. (C) Functional validation of *HAL9* mutations in lineage C (*HAL9*^A1543T^) mutant background. Assays were performed on allele-swapped mutants as described in panel A, using synthetic defined medium. Dose-response data for each strain are normalized to their respective drug-free controls and plotted using Java TreeView3; see color bar.

As a complement to investigating the mode of action of MMV688766 through lipidomics, we used an experimental evolution approach to identify mutations sufficient to confer resistance to the compound. A very amenable strain for this method has been engineered in S. cerevisiae through the disruption of 16 genes related to ABC transporter function (16ABC) (44). This sensitized background enables the selection of resistant mutants at a lower compound concentration and reduces the likelihood of efflux-related mechanisms of resistance arising (44, 45). We evolved resistance to MMV688766 by repeated passaging of independent lineages in rich liquid medium (YPD) containing increasing MMV688766 concentrations (46). Selection was terminated once cells were able to grow at an MMV688766 concentration equivalent to 16-fold (100 μM) the MIC_80_ of the parental strain (~6.25 μM). Resistant polyclonal lineages were then plated on agar containing an equivalent concentration of MMV688766 to isolate individual clones, and the resistance of these clones was confirmed through a liquid dose-response assay (Fig. 6A). Three lineages (denoted B, C, and D) demonstrated reproducible resistance to MMV688766 up to 100 μM (Fig. 6A). Examination of a representative mutant revealed that accumulation of lipid droplets upon MMV688766 exposure was greatly reduced compared to the 16ABC parent (Fig. S4C and D). To confirm that the resistance phenotype observed was not merely due to enhanced efflux activity, resistant lineages were stained with Nile red, a substrate for fungal ABC and major facilitator superfamily transporters, and accumulation of the dye was quantified by flow cytometry (47). Relative to the efflux-compromised 16ABC strain, no enhanced dye accumulation was observed across the evolved lineages, while as expected, the efflux-competent control (S. cerevisiae BY7471) showed much less dye accumulation than the 16ABC strain (Fig. S4E). To identify mutations present in the resistant lineages, strains were subjected to whole-genome sequencing using the Illumina NextSeq 500 platform. Interestingly, all three independent lineages harbored unique single nucleotide polymorphisms (SNPs) in *HAL9* (B: *HAL9*^C2214A^, Y738*; C: *HAL9*^A1543T^, K515*; and D: *HAL9*^A2479C^, S827R; Fig. 6A), which were validated using Sanger sequencing. Given the identification of premature stop codons, we hypothesized these mutations were loss of function in nature. Indeed, targeted-deletion of *HAL9* resulted in an approximately 8-fold increase in resistance in the 16ABC parent (*hal9Δ*) and did not significantly alter the MMV688766-resistance profile of the representative evolved lineage *HAL9*^A1543T^ (*hal9^A1543T^Δ*), confirming that the mutations identified in the resistant lineages caused loss of function (Fig. 6B and C).

*HAL9* encodes a zinc-cluster transcription factor that is an important determinant of ion homeostasis in yeast (48). This transcription factor has also been implicated in diverse stress responses, facilitating acid tolerance in S. cerevisiae (49), as well as acid and osmotic stress tolerance in C. glabrata (50). To determine whether the mutations identified in *HAL9* were necessary and sufficient to confer MMV688766 resistance, an allele-swap strategy was used. First, we determined whether a lack of functional *HAL9* was necessary for resistance by replacing the deleted *HAL9* allele in both the *hal9Δ* and *hal9^A1543T^Δ* strains with wild-type *HAL9* (*HAL9^WT^*) encoded on a plasmid (Fig. 6B and C). The sensitivity of both deletion strains to MMV688766 was restored upon complementation with wild-type *HAL9*, suggesting that the loss of function of *HAL9* was necessary for MMV688766 resistance (Fig. 6B and C). To determine whether the three SNPs (*HAL9*^C2214A^, *HAL9*^A1543T^, and *HAL9*^A2479C^) were sufficient to confer resistance, we again transformed *hal9Δ* and *hal9^A1543T^Δ* knockout strains with plasmids harboring *HAL9*^C2214A^, *HAL9*^A1543T^, and *HAL9*^A2479C^. In both strain backgrounds, each transformant maintained an ≥8-fold increase in resistance to MMV688766 compared to the 16ABC parent (Fig. 6B and C). This highlights that each SNP is sufficient to confer MMV688766 resistance. Therefore, the loss of function of *HAL9* enables cells to more effectively withstand stress exerted by MMV688766.

### *HAL9* loss of function increases MMV688766 resistance through upregulation of Hsp12.

**FIG 7:**
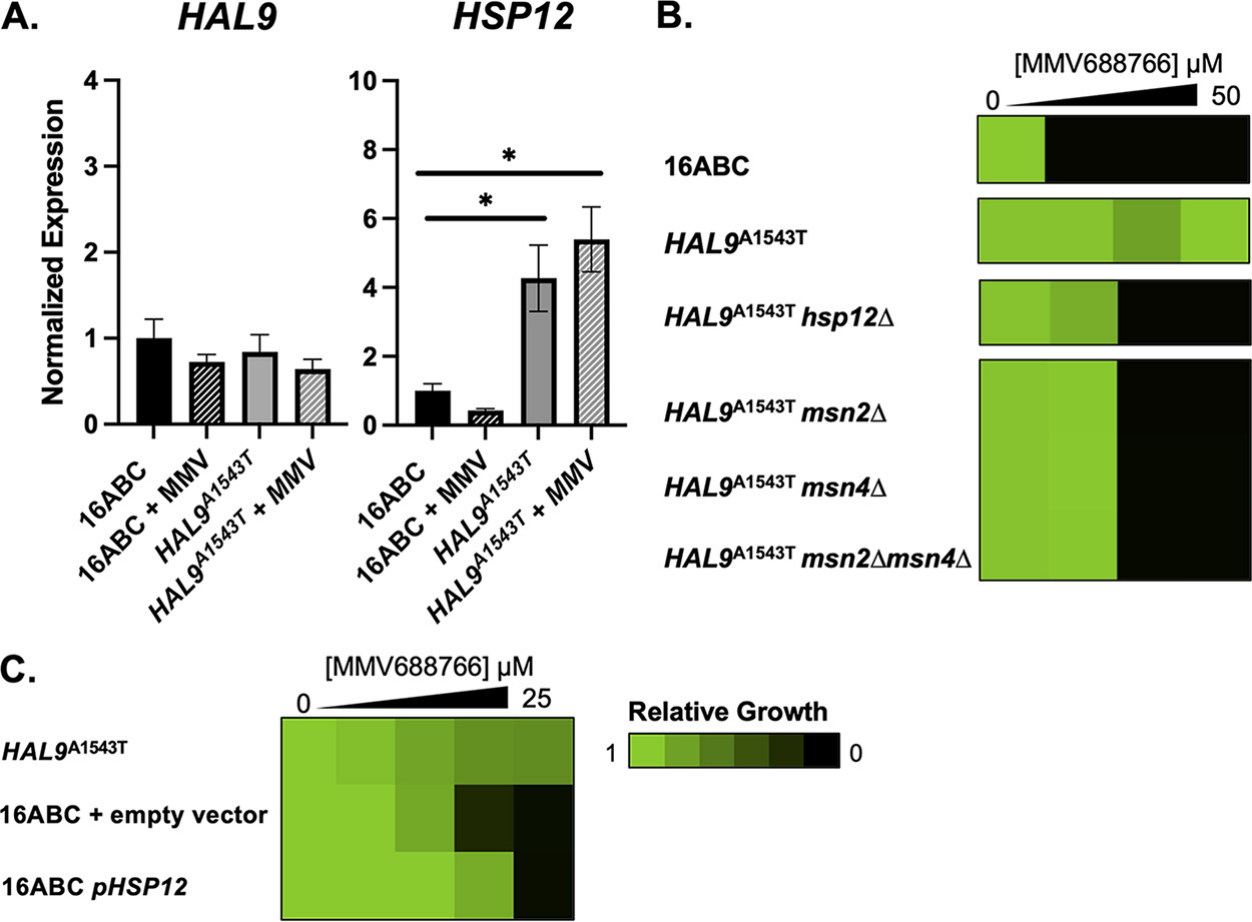
Loss of function of *HAL9* results in upregulation of stress response protein *HSP12*. (A) Transcript levels of *HAL9* and *HSP12* were measured from untreated and treated (25 μM MMV688766) 16ABC parental cells by RT-qPCR and normalized to *ACT1.* Values were made relative to untreated 16ABC control sample. Error bars represent standard error of the mean (SEM) among technical triplicates. Significance was measured using a two-tailed unpaired *t test* with Welch’s correction: *, *P < *0.05 compared to untreated. (B) Susceptibility of *hsp12Δ*, *msn2Δ*, *msn4Δ*, and *msn2Δmsn4Δ* mutants in the 16ABC and *HAL9*^A1543T^ mutant background. Each strain was assessed in 2-fold dose-response assay under shaking conditions at 30°C in YPD medium. Growth was measured after a 72-h incubation. (C) Susceptibility of *pHSP12^OE^*16ABC mutant alongside empty-vector and parental (*HAL9*^A1543T^) controls was tested in 2-fold dose-response assay under shaking conditions at 30°C in synthetic defined media (SD) to maintain plasmid selection. Growth was measured after a 72-h incubation. Growth for each strain was normalized to their respective drug-free controls and displayed using Java TreeView3; see color bar.

Moving forward, we investigated how the loss of function of *HAL9* might confer MMV688766 resistance. As *HAL9* is a transcription factor involved in response to various cellular stressors, we evaluated the expression levels of stress response-associated genes annotated in the Hal9-regulatory pathway (48, 51). These experiments were performed in the 16ABC parent and the *HAL9*^A1543T^ background as a representative mutant lineage, in the presence and absence of MMV688766. Interestingly, expression levels of *HAL9* were virtually unchanged under all conditions tested, as was the expression of *ENA1*, a canonical target of Hal9 (Fig. 7A and Fig. S5A). However, *HSP12*, a small heat shock protein that stabilizes and modulates fluidity of the yeast cell membrane under diverse stress conditions (52, 53), was significantly overexpressed in the *HAL9*^A1543T^ mutant in the presence and absence of MMV688766 compared to the 16ABC parent (Fig. 7A). This result suggests overexpression of *HSP12* may contribute to the MMV688766-resistance phenotype.

10.1128/mbio.02730-22.5 FIG S5 Loss of function of *HAL9* does not significantly alter the expression of *ENA1*, 16ABC p*HSP12* strain overexpresses *HSP12*. (A) Transcript levels of *ENA1* in treated (25 μM MMV688766) and untreated 16ABC parental and *HAL9*^A1543T^ mutant cells were measured by RT-qPCR and normalized to *ACT1.* Values are relative to the untreated 16ABC control sample. (B) Transcript levels of *HSP12* in the 16ABC and 16ABC+p*HSP12* transformed strains were measured by RT-qPCR and normalized to *ACT1, ALG9, TAF10*, and *UBC6*. Error bars represent standard error of the mean (SEM) among technical triplicates. Significance was measured using a two-tailed unpaired *t* test with Welch’s correction: **, *P < *0.005. (C) Pseudocolored density plots displaying the side-scatter and forward-scatter data for each event recorded in an untreated sample of C. auris (CDC0387) and S. cerevisiae (BY4741) when run on a Cytoflex flow cytometer, analyzed with CytExpert Software. Download FIG S5, PDF file, 0.4 MB. Copyright © 2022 Puumala et al. 2022 Puumala et al. https://creativecommons.org/licenses/by/4.0/ This content is distributed under the terms of the Creative Commons Attribution 4.0 International license.

**FIG 8:**
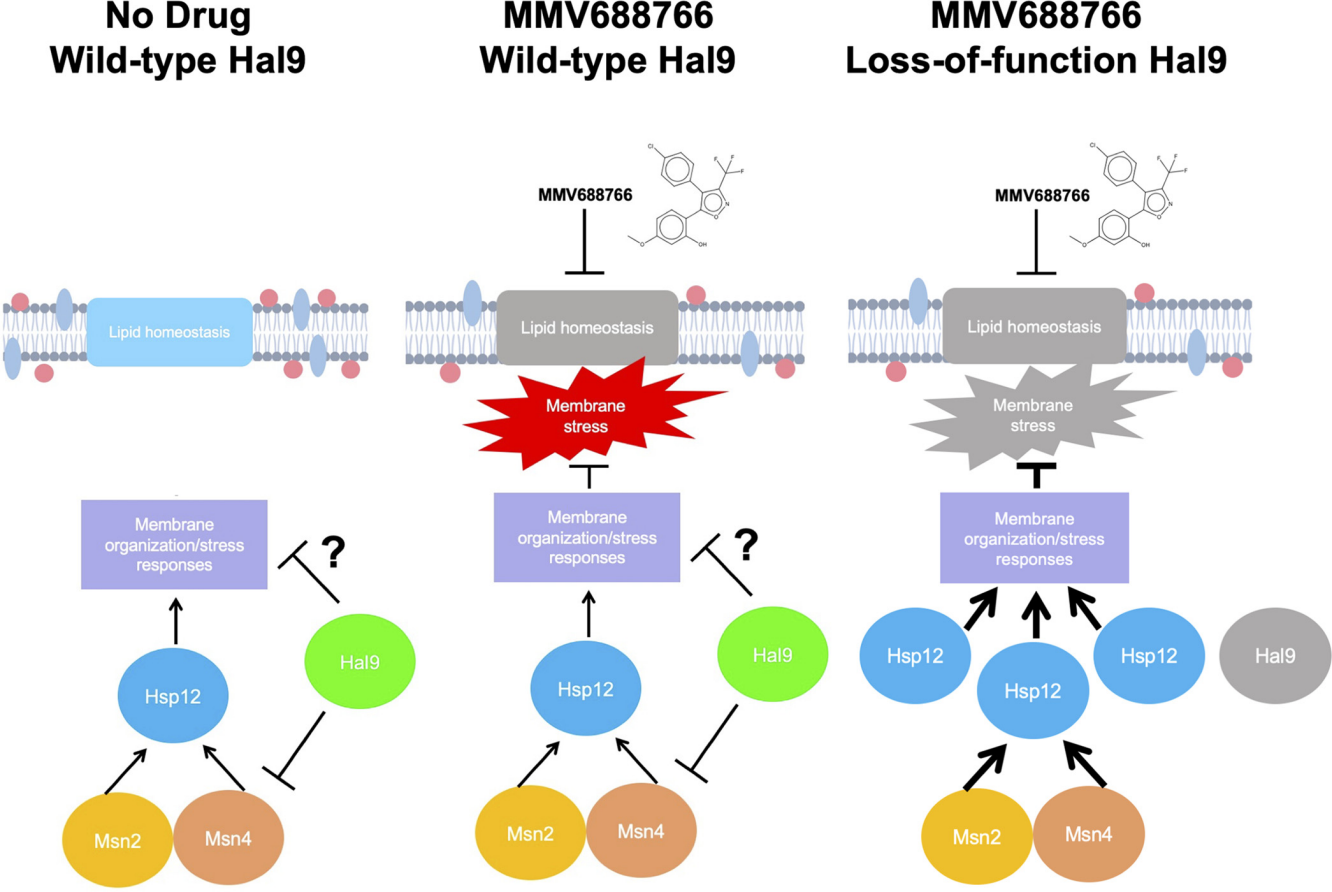
Schematic representing a model detailing how Hal9 governs MMV688766 susceptibility in S. cerevisiae. Under normal physiological conditions, Hal9 regulates the activity of Msn2, Msn4, Hsp12, and unknown regulators to regulate membrane organization and membrane stress responses. MMV688766 exposure impairs lipid homeostasis leading to the induction of membrane stress. When Hal9 function is impaired, increased signaling through Msn2 and Msn4 drives the upregulation of Hsp12, which activates key membrane stress responses important for compound-induced membrane stress.

Finally, we sought to determine whether the loss of *HAL9* function facilitates MMV688766 resistance, either through direct upregulation of *HSP12* or indirectly through another regulator. Notably, the expression of *HSP12* is positively regulated by the partially redundant transcriptional regulators Msn2 and Msn4 (54), with both proteins able to recognize and bind stress-response elements in the *HSP12* promoter (54–56). To assess genetically whether Msn2, Msn4, and/or Hsp12 play a role in MMV688766 susceptibility, we generated a set of mutants in the 16ABC and *HAL9*^A1543T^ backgrounds. Specifically, we deleted *HSP12*, *MSN2*, *MSN4*, as well as *MSN2* and *MSN4* in combination in a representative resistant lineage to determine whether the loss of any of these genes was sufficient to confer enhanced sensitivity to MMV688766. Dose-response testing revealed that *hsp12Δ*, *msn2Δ*, *msn4Δ*, and *msn2Δmsn4Δ* strains in the *HAL9*^A1543T^ background demonstrated increased sensitivity to MMV688766 in comparison to the *HAL9*^A1543T^ strain, but all mutants were still 2-fold more resistant than the 16ABC parent (Fig. 7B). Reciprocally, we generated an *HSP12* overexpression strain to test whether *HSP12* overexpression alone is sufficient to confer MMV688766 resistance. Wild-type *HSP12* was cloned into an expression vector under the control of a constitutive GPD promoter (p*HSP12*^OE^) and transformed into the 16ABC parent. A 60-fold increase in *HSP12* expression was documented by reverse transcription-quantitative PCR (RT-qPCR) (Fig. S5B). Compared to the empty-vector control strain, we observed a 2-fold increase in MMV688766 resistance, only a partial restoration of the level of resistance seen in the *HAL9*^A1543T^ strain (Fig. 7C). Collectively, these results suggest a model in which the resistance to MMV688766 in S. cerevisiae conferred by *HAL9* loss of function is in part mediated through activation of Msn2/4, which then drives increased expression of *HSP12*, an important regulator of membrane fluidity and membrane lipid homeostasis (Fig. 8) (53).

## DISCUSSION

The unprecedented antifungal-resistance profiles and high disease mortality rates observed across C. auris isolates pose an urgent threat to global human health (57). Prompt and sustained efforts are imperative to equip clinicians and public health officials with effective strategies to combat multidrug-resistant C. auris infections. In this study, we demonstrate that existing libraries of drug-like chemical matter can serve as rich resources for molecular scaffolds with novel antifungal activity. Specifically, by screening the MMV Pathogen Box library, we uncovered a trisubstituted isoxazole, MMV688766, with previously undescribed fungicidal activity that through chemogenomic, metabolomic, and phenotypic analyses was determined to disrupt fungal lipid homeostasis. Moreover, efforts to further define the mode of action of MMV688766 revealed a role for the transcription factor Hal9 in the response of S. cerevisiae to altered lipid homeostasis, which involves the upregulation of *HSP12*. Thus, this work identifies and characterizes a compound with novel fungicidal activity and unveils genetic circuitry by which loss of function of the transcription factor Hal9 enables tolerance of drug-induced stress.

Antifungal discovery remains a considerable challenge given the fact that bioactive molecules must be able to traverse the fungal cell wall and membrane, as well as evade complex efflux mechanisms. As an approach to overcoming the challenge, this work highlights the power of leveraging existing compound collections to identify scaffolds with novel antifungal activity against drug-resistant pathogens like C. auris (30). Although the Pathogen Box encompasses a relatively limited collection of small molecules, these compounds were selected from a set of ~4 million compounds due to their low toxicity against mammalian cells and favorable activity against specific microbial pathogens (https://www.mmv.org/mmv-open/pathogen-box/about-pathogen-box). Alongside prior screens of this chemical resource, we confirmed that molecules active against causative agents of neglected tropical diseases can display parallel activity against diverse fungal pathogens. For example, MMV688768, MMV687273, and MMV687807 have all been reported to reduce the metabolic activity of C. albicans biofilms (29), and MMV688271 was identified as a potent inhibitor of C. albicans and C. neoformans growth in nutrient-limited conditions (27).

Here, we show that treatment with MMV688766 results in an impairment of fatty acid biosynthesis. Although the specific molecular target(s) of the compound remain unknown, it appears to compromise *de novo* synthesis at an early stage in a novel manner that does not involve direct inhibition of FAS. The apparent elusiveness of a direct protein target for MMV688766 may also be explained by a general, membrane-perturbing mode of action. A recent investigation of 3,4,5-trisubstituted isoxazoles for the inhibition of Leishmania amazonensis revealed that treatment with derivatives of this scaffold is associated with plasma membrane rigidity in the parasite (58). These findings also supported a potential peroxidation of membrane proteins and lipids in the plasma membrane (58). As MMV688766 has a similar trisubsitituted isoxazole scaffold, employing a 4-chlorophenyl group distinct from an aryl imine substitution used by the molecules previously studied (58), it is plausible that this molecule perturbs fungal growth through membrane disruption, thereby giving rise to the broad alterations in lipid homeostasis observed in response to MMV688766 throughout this study.

Considering lipid inhibition independent of MMV688766, major success has been achieved in the development of drugs that target microbial lipid homeostasis, including the commonly deployed azole and polyene antifungal classes (2, 40, 59). These compounds exploit the evolutionary divergence between mammalian and fungal membrane sterols. However, considerable divergence in other lipid metabolic pathways exists between fungi and humans that has yet to be fully exploited. Given the importance of fatty acid synthesis across all life forms, FAS has emerged as an important target for the development of compounds with a range of antimicrobial activities (60–62), and despite the fact that this complex is conserved in humans, it has been suggested that fungal-selective FAS inhibitors could be identified (63). Supporting this notion is the fact that the fatty acid biosynthesis inhibitor isoniazid remains an important treatment for Mycobacterium tuberculosis infection (64). Furthermore, as sphingolipids are key players in growth, morphogenesis, and virulence in fungal pathogens (40), fungal-selective inhibition of this pathway may also show therapeutic promise (65). Supporting this idea is the fact that natural-product sphingolipid biosynthesis inhibitors aureobasidin A, galbonolide A, and khafrefungin, as well as synthetic molecules *N*′-(3-bromo-2-hydroxybenzylidene-2-methylbenzohydrazide (BHBM) and 3-bromo-*N*′-(3-bromo-4-hydroxybenzylidene)benzohydrazide (D0), all demonstrate potent and selective antifungal activity against diverse fungal pathogens (65–68).

Beyond the value of identifying chemical scaffolds with antifungal activity, this work emphasizes how small molecules can be used to probe the biology of diverse fungi and investigate how stress response mechanisms enable microbes to survive in the presence of antifungal assault. Throughout this study, we aimed to evolve mutants that were resistant to MMV688766 to develop further insight into its mechanism of action (45, 69). While experimental evolution can reveal the target of small molecules (45), it also provides insight into signaling pathways involved in the cellular responses to drug-induced stress (69). A very clear signature emerged from our study, implicating the understudied, nonessential zinc-cluster transcription factor *HAL9* in the S. cerevisiae response to MMV688766. Based on our findings, we hypothesize that the absence of Hal9 preactivates the Msn2/4 response and upregulates Hsp12 levels giving the cells the ability to survive the subsequent challenge by MMV688766. This is similar to how in the well-conserved phenomenon of thermotolerance, prior nontoxic heat stress enables cells to tolerate subsequent, otherwise lethal heat stress (70, 71). A recent large-scale investigation of adaptive evolution to xenobiotics revealed that SNPs in *HAL9* confer resistance to other molecules, specifically loratadine and MMV085203, in the same S. cerevisiae drug-sensitized background that was used in this work (46). A premature stop codon that confers resistance to the antimalarial napthoquinolone derivative MMV085203 (*HAL9*^C1542A^, Y514*) maps very closely to the mutation we identified in one of the MMV688766-resistant backgrounds (*HAL9*^A1543T,^ K515*) (46, 72). The precise mechanism of action of MMV085203 has not been defined; however, it is predicted to target the plasmodial mitochondria (72). Interestingly, this suggests that while loss of function of *HAL9* in S. cerevisiae and C. glabrata is associated with decreased resistance to pH, halogen, and osmotic stressors, it is conversely associated with increased resistance to xenoibiotics in S. cerevisiae, including sphingolipid biosynthesis inhibitors (46, 48–50). Further research is needed to gain a complete understanding of the S. cerevisiae *HAL9* regulatory network, its links to drug resistance, and if and how this network governs compound susceptibility in diverse fungal pathogens. Likewise, the expression and regulation of Hsp12, as it relates to drug resistance in diverse pathogenic fungi, represent interesting avenues of future study, particularly as lipid biosynthesis inhibitors demonstrate therapeutic promise as antifungals. Overall, a comprehensive understanding of drug-resistance mechanisms is crucial in drug development efforts seeking to identify resistance-evasive therapeutic strategies.

## MATERIALS AND METHODS

### Fungal strains and culture conditions.

Archives of all strains were maintained at −80°C in 25% glycerol. Strains were grown in standard conditions at 30°C in YPD (1% yeast extract, 2% peptone, 2% dextrose), in RPMI (10.4 g/L RPMI powder with l-glutamine [Gibco], 165 mM MOPS, 2% glucose, 5 mg/mL histidine, pH 7), or SD (2% glucose, 6.7 g/L yeast nitrogen base without amino acids). All strains used in this study are listed in Table S1. All plasmids used in this study are listed in Table S2. All oligonucleotide sequences used in this study are listed in Table S3. For all mutants generated for this study, relevant strain and plasmid construction details are included in Text S1.

10.1128/mbio.02730-22.7 TEXT S1 Supplemental methods and references. Download Text S1, PDF file, 0.1 MB. Copyright © 2022 Puumala et al. 2022 Puumala et al. https://creativecommons.org/licenses/by/4.0/ This content is distributed under the terms of the Creative Commons Attribution 4.0 International license.

10.1128/mbio.02730-22.7 TABLE S1 Strains used in this study. Download Table S1, PDF file, 0.1 MB. Copyright © 2022 Puumala et al. 2022 Puumala et al. https://creativecommons.org/licenses/by/4.0/ This content is distributed under the terms of the Creative Commons Attribution 4.0 International license.

10.1128/mbio.02730-22.8 TABLE S2 Plasmids used in this study. Download Table S2, PDF file, 0.1 MB. Copyright © 2022 Puumala et al. 2022 Puumala et al. https://creativecommons.org/licenses/by/4.0/ This content is distributed under the terms of the Creative Commons Attribution 4.0 International license.

10.1128/mbio.02730-22.9 TABLE S3 Oligonucleotides used in this study. Download Table S3, PDF file, 0.2 MB. Copyright © 2022 Puumala et al. 2022 Puumala et al. https://creativecommons.org/licenses/by/4.0/ This content is distributed under the terms of the Creative Commons Attribution 4.0 International license.

### Pathogen Box chemical screen.

The Pathogen Box library, consisting of five 96-well microtiter plates, was screened at a final concentration of 25 μM against a C. auris clinical isolate (CDC0387). An overnight culture of CDC0387 was diluted to an OD_600_ of 0.00015 in RPMI medium in 96-well microtiter plates at a final volume of 0.2 mL/well. Plates were incubated at 30°C under static conditions in the dark, and growth was measured by optical density (OD_600_) after 48 h using a spectrophotometer (Molecular Devices). Screen hits were defined as inhibiting growth by at least 80% relative to no drug controls.

### Dose-response assays.

Drug susceptibility assays were performed in 384-well plates in a final volume of 0.04 mL/well with 2-fold dilutions of each compound in RPMI or YPD medium, as indicated. Plates were incubated in the dark at 30°C or 35°C under static conditions, and OD_600_ was measured after the indicated incubation times using a spectrophotometer (Molecular Devices). Data were quantitatively displayed as heat maps using Java TreeView 1.1.6. Note that CLSI conditions for broth microdilution assays were used if indicated, following M27-A3/S4 (32). In assays using A. fumigatus, relative viable cell number was quantified by incubating cells with alamarBlue (0.03% wt/vol) after a 48-h incubation followed by an additional 24-h incubation at 37°C. Growth of A. fumigatus was also observed microscopically using an IncuCyte live cell imager after a 48-h incubation with compound at ×20 magnification (scale bar = 200 μm). For assays employing strains from the GRACE collection, strains were grown overnight in the presence and absence of the indicated DOX concentration(s). In lipid-supplemented dose-response assays, the indicated fatty acids and sphingolipid intermediates were prepared at a 10% concentration in DMSO and added to the fungal inoculum prepared in YPD medium to a final concentration as indicated. When resistant mutants evolved through liquid selection were tested, 2-fold dose-response assays were performed under shaking conditions in 2-mL volumes of a culture of YPD or SD medium as indicated. Tubes were incubated in the dark at 30°C, and OD_600_ was measured after the indicated incubation times using a spectrophotometer (Molecular Devices).

### Mammalian cytotoxicity assays.

HepG2 cells (cat. no. HB-8065; ATCC) were counted using a hemocytometer and diluted to 5× 10^4^ cells/mL in 100 μL of RPMI 1640 (Sigma) medium supplemented with 10% heat-inactivated fetal bovine serum (Gibco). Cells were seeded in black, clear-bottom 384-well plates (Corning) to a final density of 2,000 cells/well in 40 μL. Cells were incubated at 37°C with 5% CO_2_ for 24 h. Subsequently, a 2-fold dilution series of test compound was added to seeded cells from 0 μM to 100 μM, and plates were incubated at 37°C with 5% CO_2_ for 72 h. After 72 h, alamarBlue (Invitrogen) was added to the HepG2 cells at a final concentration of 0.5× and plates were incubated at 37°C for 4 h. Fluorescence was measured at excitation/emission of 560/590 nm using a TECAN Infinite F200Pro microplate fluorometer and values were corrected for background from the medium. All assays were performed in technical triplicates and in at least two biological replicates. Relative viability was calculated as a percentage by dividing corrected fluorescence in the treatment wells by that measured in the corresponding DMSO control wells, multiplying by 100%.

### C. elegans infection assays.

C. elegans strain *glp-4* was obtained from the *Caenorhabiditis* Genetics Center (CGC) and maintained on superfood nematode growth media (NGM) with E. coli (OP50) at 15°C, as previously described (73). After 5 days, worms were transferred to 25°C for 2 days to synchronize growth. A culture of C. albicans SC5134 in YPD was incubated overnight at 30°C under shaking conditions. Infection plates were made by plating 10 μL of the overnight culture onto brain heart infusion (BHI) agar plates containing 50 μg/mL kanamycin. Plates were incubated at 30°C for 16 h to create fungal lawns. A liquid media-based C. elegans infection assay protocol was used, based on previous studies (74–76). Briefly, 48-well plates were prepared with each well containing either MMV688766 or no drug treatment in infection medium (80% M9 buffer, 20% BHI, 10 μg/mL cholesterol, and 90 μg/mL kanamycin). Synchronized NGM plates containing C. elegans were washed twice with 6 mL of M9 buffer with swirling to dislodge the worms and transferred to a 15-mL conical tube. Worms were allowed to sediment in the conical tube for 5 to 10 min before the supernatant was removed and replaced with fresh M9 buffer, three times. Conical tubes were centrifuged at 1,500 × *g* for 40 s, and buffer was removed and fresh M9 buffer was added. The worms were counted under a stereomicroscope at ×10 magnificatio,n and the concentration of worms per microliter of the solution was calculated, as previously described (73). The worm solution was adjusted to 1 worm/μL, and 100 worms were added to BHI + kanamycin plates in the absence or presence of C. albicans and allowed to feed for 3 h. Worms were then rinsed off the plate with 500 μL of M9 buffer and transferred to a 15-mL conical tube, and 10 mL of additional M9 buffer was added. The worms were washed by allowing to sediment and centrifuging, as previously described. The worm pellet was suspended in 300 μL of M9 and worms were allowed to crawl on unseeded BHI plates for 30 min. Worms were rinsed from the plates with 1 mL of M9 buffer and transferred into a new conical tube. The concentration of worms per microliter was determined again, and the solution was adjusted to 1 worm/μL. Twenty-five worms (25 μL of solution) were added to each well of the prepared 48-well plates. Plates were incubated at 25°C and worm survival was scored at 24 h under a stereomicroscope at ×10 magnification.

### Haploinsufficiency profiling.

Glycerol stock pools of heterozygous (HET) double-barcoded deletion mutants were thawed, diluted to an OD_600_ of 0.05 into triplicate 60 mL YPD cultures, and grown at 30°C under shaking conditions for 90 min. Subsequently, the three cultures were each diluted 2-fold in a final volume of 5 mL YPD in the presence or absence of 8 μM MMV688766 and grown at 30°C under shaking conditions for 18 h. Cultures were harvested by centrifugation and cell pellets were stored at −80°C. Samples were prepared for sequencing as previously described (77). UP-TAG and DOWN-TAG primer sequences are included in Table S3. Equal quantities of UP-TAG and DOWN-TAG pools were combined to form a sequencing library, which was sequenced on an Illumina NextSeq500 instrument (Mid-Output, V2 Chemistry) using specific primers to sequence and index the UP-TAGs and DOWN-TAGs (Table S3). Barcode sequence reads were mapped to an artificial genome containing known UP-TAG and DOWN-TAG sequences using Bowtie v1.0 (http://bowtie-bio.sourceforge.net/index.shtml). Read frequencies for the UP-TAG and DOWN-TAG of each strain were compiled for each indexed sample. UP-TAGs or DOWN-TAGs for which more than one of the triplicate samples for solvent-only read counts were <20% of the median read per million mapped reads were omitted from further analysis. Log_2_-fold differences for each strain UP-TAG and DOWN-TAG were calculated. Strains were considered significantly reduced in frequency if the log_2_ solvent:drug ratio was ≥5 MADs above the median in both the UP-TAG and DOWN-TAG or if one of the UP-TAG and DOWN-TAG was ≥5 MAD above the median and the opposing TAG was emitted due to low reads. MAD upstream barcodes were 0.23, MAD downstream barcodes were 0.23, median upstream barcodes were 0.024, and median downstream barcodes were 0.204 (77).

### Fungal growth curves.

Strains were grown in YPD medium overnight and diluted to an OD_600_ of 0.00015 in YPD medium in 384-well plates in a final volume of 0.04 mL. Plates were grown at 30°C under shaking conditions, and OD_600_ was measured every 15 min for 48 h using a 12-microplate cycling robot (S&P Robotics) that employs a Multiskan FC microplate photometer (Thermo Fisher) for OD_600_ measurements. Data were analyzed in GraphPad Prism 9. For HIP verifications, the growth of heterozygous deletion strains was quantified by calculating the ratio of the area under the curve (AUC) in the presence of 8 μM MMV688766 relative to the AUC in drug-free medium (YPD).

### Lipid droplet assays.

Fungal cells were grown to saturation overnight in 5 mL YPD medium under shaking conditions at 30°C. Cells were then subcultured to OD_600_ ~0.1 in 2 mL YPD medium and grown for 2 h under shaking conditions at 30°C. Next, compounds were added to subcultured tubes, and cells were allowed to grow for a further 4 h under the same conditions. Cells were then stained with 1 μg/mL BODIPY 493/503 for 10 min. One milliliter of each stained cell sample was centrifuged at 11,200 × *g* for 1 min. The pellet was resuspended in 1 mL phosphate-buffered saline (Thermo Fisher) and centrifuged for 1 min at 11,200 × *g*. This process was repeated twice before each sample was visualized on Zeiss Axio Imager.MI at ×100 magnification using differential interference contrast (DIC) and enhanced green fluorescent protein (EGFP) fluorescence filter.

Flow cytometry was performed to quantify relative lipid droplet volume in each treated cell population using a CytoFLEX LX flow cytometer (Beckman Coulter). Cells were analyzed for incorporation of BODIPY 493/503 using the FITC (488 nm) channel, and intact cells were gated from debris after comparing forward versus side scatter (Fig. S5C). Distributions of FITC fluorescence intensity of each population were collected with median FITC-A values from >20,000 measurement events.

### Lipidomics sample preparation.

Cell pellets were resuspended in 150 μL of water and subjected to three cycles of freeze and thaw and then sonicated for 10 min. To analyze polar lipids/metabolites samples, 600 μL of 1:1 μL of methanol and water was added to each sample and vortex for 1 min. To analyze nonpolar lipids, 600 μL of 1:2 chloroform:methanol was vortexed for 1 min. Both polar and nonpolar lipid samples were spun at max speed in a microcentrifuge for 10 min. Supernatant of the polar lipid extract was collected in a fresh tube, and the bottom layer of the nonpolar extract was collected. Next, polar and nonpolar lipid extracts were dried completely under nitrogen gas using a turbovap (Zymark). Dried samples were reconstituted in liquid chromatography-mass spectrometry (LC-MS) grade water or in LC-MS grade 9:1 chloroform methanol. Sample concentrations were normalized by the optical density of cells measured at sample collection. All samples were stored at −80°C until analysis on LC-MS. Samples prepared from stable isotope labeling were prepared for polar lipid measurement only.

### Reverse-phase LC-MS for nonpolar lipids.

Nonpolar lipid samples were analyzed using two LC-MS methods. In positive-ion mode mode, lipids were injected onto an Elute II LC system using a Waters Acquity UPLC CSH C_18_ column (130 Å, 1.7 μm, 2.1 mm × 100 mm) with a starting gradient of 60% buffer A (60:40 acetonitrile:water + 10 mM ammonium formate) and 40% buffer B (90:10 isopropanol:acetonitrile + 10 mM ammonium formate). In negative-ion mode, the mobile phase contained 0.1% formic acid in place of the 10 mM ammonium formate. The initial flow rate was 0.4 mL/min, and the gradient was held for 2 min at 40% then increased to 99% B for 12 min. Then, 99% B was held for 6 min and decreased rapidly back to 40% and regenerated for 2 min. Lipids with mass to charge between 100 and 1,350 were detected in a TimsToF PRO (Bruker) running with Tims device ON and collecting Parallel accumulation-serial fragmentation (PASEF) data with ion source conditions as follows: Drying gas was heated to 200°C at a flow of 10 L/min, Nebulizer gas was 200°C and 40 lb/in^2^, and capillary voltage was 2500 V. Default values for PASEF and Tims device parameters were used.

### Ion-paired reverse-phase LC-MS for polar lipids.

Metabolite samples were injected onto an infinity 1290 UPLC system outfitted with an Agilent ZORBAX Extend-C_18_ column (1.8 μm, 2.1 mm × 150 mm) with starting conditions of 99% buffer A (97:3 water:methanol + 10 mM TBA and 15 mM acetic acid) and 1% buffer B (methanol) and a flow rate of 0.25 mL/min. The starting gradient was held for 2.5 min then increased to 20% B over the next 5 min, then increased again to 45% B over 5.5 more minutes, and finally to 99% B over 2 min. The column was purged for 5 more minutes at 99% B and then regenerated at 1% B for 2 min. To minimize carryover and increase column, life a second binary pump is used between runs to clean the column using buffer A as described above and 100% acetonitrile as buffer B.

Polar lipids were detected using a 6550 AJS-ESI-IFunnel QToF mass spectrometer (Agilent) running in full-scan MS1 mode. The ion source conditions were as follows: nebulizer sheath gas was heated to 325°C and gas flow was 12 L/min. Nebulizer gas temperature was 150°C and gas flow as 14 L/min at 45 lb/in^2^g. Capillary and nozzle voltages were set to 2,000 V. iFunnel voltages were set to −30 V DC, high-pressure funnel drop −100 V and radio frequency (RF) voltage of 110 V, and low-pressure funnel drop −50 V and RF voltage of 60 V.

### Metabolite identification and quantitation.

Nonpolar lipids were identified and annotated using exact mass and MS2 spectrum matching to the database via the Metaboscape software (Bruker). Polar lipids/metabolites were annotated using exact mass and retention time to match to database previously constructed using neat standards. Metabolite levels were determined by integrating the area under the curve of the molecular ion using Metaboscape. Next, significantly altered metabolites were identified by comparing the mean measurement in treated versus untreated samples and evaluating the means using a two-tailed *t* test. Only metabolites with a *P* value of <0.1 were kept for further analysis. For figure generation, integrated areas were centered around the median for each metabolite and log_2_ transformed.

### Stable isotope tracing.

Stable isotope tracing samples were analyzed using the ion-paired reverse-phase LC-MS method for polar lipids. ^13^C enrichment in palmitic acid, oleic acid, and linoleic acid was determined by extracting [M-H] masses of each theoretical isotopolog starting with uniform ^12^C atoms composition and iteratively, adding one neutron until uniformly labeled with ^13^C atoms. Intensities were then corrected to remove the ^13^C intensity expected to be present naturally using the IsoCorrectoRGUI R package (https://www.bioconductor.org/packages/release/bioc/html/IsoCorrectoRGUI.html). The proportion of ^13^C enrichment was calculated by dividing the intensity of each isotopolog by the sum of intensities of all isotopologs for each metabolite.

### FAS purification and activity assay.

FAS complex was purified from S. cerevisiae (JWL01) and C. albicans (CaLC5425) as described in reference 41. One minor deviation was that C. albicans was grown in 500 mL YPD medium in 2-L flasks under shaking conditions (Thermo Electron) at 30°C for ~5 h in the presence or absence of 25 μM MMV688766. Growth medium was supplemented with 0.015% each of palmitic, myristic, and oleic acids, respectively, to compensate for growth inhibition due to compound treatment. The activity of purified FAS at 12 μg per well was measured through a spectrophotometric assay, adapted from reference 78. The reaction buffer contained 200 mM potassium phosphate pH 7.3, 417 μM acetyl-CoA (Sigma-Aldrich), 250 μM NADPH, 1.75 mM DTT, and 0.6% wt/vol BSA in a 120-μL reaction volume. The absorbance at 334 to 340 nm was measured for 5 min before 500 μM malonyl-CoA (Sigma-Aldrich) was added to each assay well and monitored for ~15 min at 25°C. As a control, DMSO was added to 1 to 1.09% vol/vol to match the highest DMSO concentration tested in the inhibitory reactions (i.e., 50 μM MMV688766). Each curve was normalized against its first point of measurement after the addition of malonyl-CoA.

### Blue native PAGE.

FAS complex was purified from C. albicans (CaLC5425) as described previously (41). C. albicans was grown under shaking conditions (Thermo Electron) in 10 mL YPD in glass tubes at 30°C for ~5 h in the presence and absence of 25 μM MMV688766. Purified samples and relevant controls were run on a NativePAGE Novex 3 to 12% BisTris gel (Life Technologies) using 50 mM BisTris, 50 mM Tricine pH 6.8 anode buffer; and 50 mM BisTris, 50 mM Tricine, 0.02% Coomassie G-250 cathode buffer in the XCell *SureLock* Mini-Cell (Life Technologies) for 90 to 115 min at 150 V. Subsequently, the gel was destained in 8% acetic acid for 10 to 15 min, washed in deionized water (~30 min), and imaged on a ChemiDoc gel imaging system (Bio-Rad).

### Evolution of resistant mutants.

Three independent lineages of S. cerevisiae ABC16 were grown overnight in 3 mL YPD medium. Each lineage was subcultured into a further 3 mL YPD to an OD_600_ of 0.1 in YPD +/− MMV688766 (6.25 μM starting concentration). Each selection culture was grown under vigorous shaking (220 rpm). Upon reaching saturation, cultures were diluted into fresh YPD (OD_600_ = 0.1) containing increasing compound concentrations (increased concentration by ~5 μM each round) (69). Once cultures reached saturation in 150 μM MMV688766, samples were frozen down in 25% glycerol. To confirm the resistance phenotype, samples were struck onto YPD agar plates containing 100 μM MMV688766. Single colonies were isolated, and a 5-point dose-response assay (in biological duplicates) with 2-fold dilutions was performed under the same shaking conditions as were performed during the resistance evolution steps to determine the MIC_80_ values of the evolved versus parental strains. To verify that resistance was not achieved through upregulation in drug efflux, Nile Red accumulation assays were performed using wild-type S. cerevisiae (BY4741), ABC16, and all resistant mutants as described in reference 47. All lineages with sufficient resistance and no significant upregulation in efflux were prepared for whole-genome sequencing.

### Sequencing analysis.

Saturated overnight cultures of the ABC16 and MMV688766-resistant mutants in each background were grown in 5 mL of YPD, and 1 mL of each was pelleted at ~16,100 × *g* for 2 min followed by flash freezing with liquid nitrogen. Genomic DNA extraction, sequencing library preparation, and analysis were performed as previously described in reference 45 using Illumina NextSeq 500, HO, 300c. Reads were aligned to the S. cerevisiae S288C reference genome. MuTect (Version 1.1.4) (79) was used to identify unique mutations in MMV688766-resistant strains. Mean genome coverages for all whole-genome sequenced strains were recorded as 16ABC: 179.46, Lineage B *HAL9*^C2214A^: 179.39, Lineage C *HAL9*^A1543T^: 159.89, Lineage D *HAL9*^A2479C^: 147.72.

Mutations identified in *HAL9* by whole-genome sequencing were validated by Sanger sequencing. Each locus or region flanking mutation(s) was PCR amplified from the genomic DNA (gDNA) of the respective resistant strains. Reaction mixtures contained 1× Q5 PCR buffer, 0.2 mM dNTPs, 0.5 μM primers, 1 unit of Q5 polymerase, ~100 ng of gDNA, and sterile water up to 25 μL. Cycling conditions were 94°C, 5 min; 95°C, 30 s; 52°C, 45 s; and 72°C, 1 min for 30 cycles; 72°C 10 min. PCR products were visually confirmed by gel electrophoresis and purified using a PCR cleanup kit (Sigma). Approximately 100 ng of purified product with ~0.625 μM appropriate oligonucleotides were sent for sequencing at the TCAG Sequencing Facility (SickKids). All primer sequences used are listed in Table S3.

### Quantitative RT-PCR.

Relevant cultures were grown overnight in YPD at 30°C. For assays assessing gene expression in the presence and absence of MMV688766, overnight cultures were diluted to OD_600_ ~0.2 and grown for 2 h of shaking at 30°C. Subcultures were then treated as indicated with or without 25 μg/mL of MMV688766 and grown at 30°C and shaken for a further 4 h. Assays independent of MMV688766 were completed by subculturing overnight cultures diluted to OD_600_ ~0.1 and 4 h of shaking at 30°C. Cultures were pelleted and frozen at −80°C. RNA was isolated using the Qiagen RNeasy kit and further treated with RNase-free DNase (Qiagen). The iScript cDNA Synthesis kit (Bio-Rad) was used to perform cDNA synthesis. PCR was performed using Fast SYBR green Master Mix (Thermo Fisher) and a Bio-Rad CFX384 Real Time System, with the following cycling conditions: 95°C for 3 min; 95°C for 10 s; and 60°C for 30 s, for 40 cycles. RT-qPCRs were performed in triplicate for biological duplicate experiments. All primer sequences are listed in Table S3.
