# Supplementary figures and images for: The Trisubstituted Isoxazole MMV688766 Exerts Broad-Spectrum Activity against Drug-Resistant Fungal Pathogens through Inhibition of Lipid Homeostasis
Source: mBio. 2022 Oct 27;13(6):e02730-22. doi: 10.1128/mbio.02730-22 (PMC9765174; doi:10.1128/mbio.02730-22)

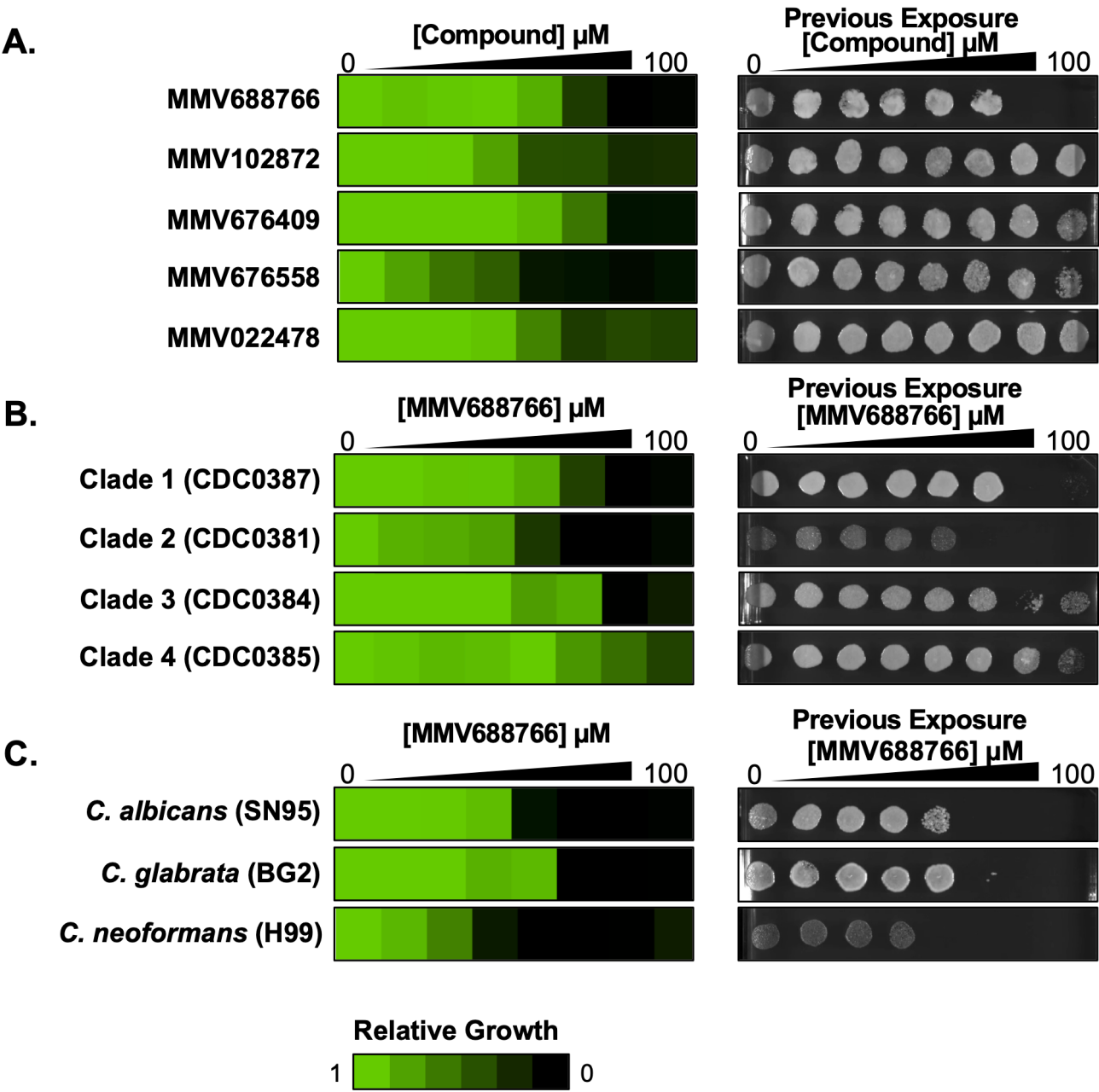

Supplement: FIG S1 [file mbio.02730-22-s0001.pdf]

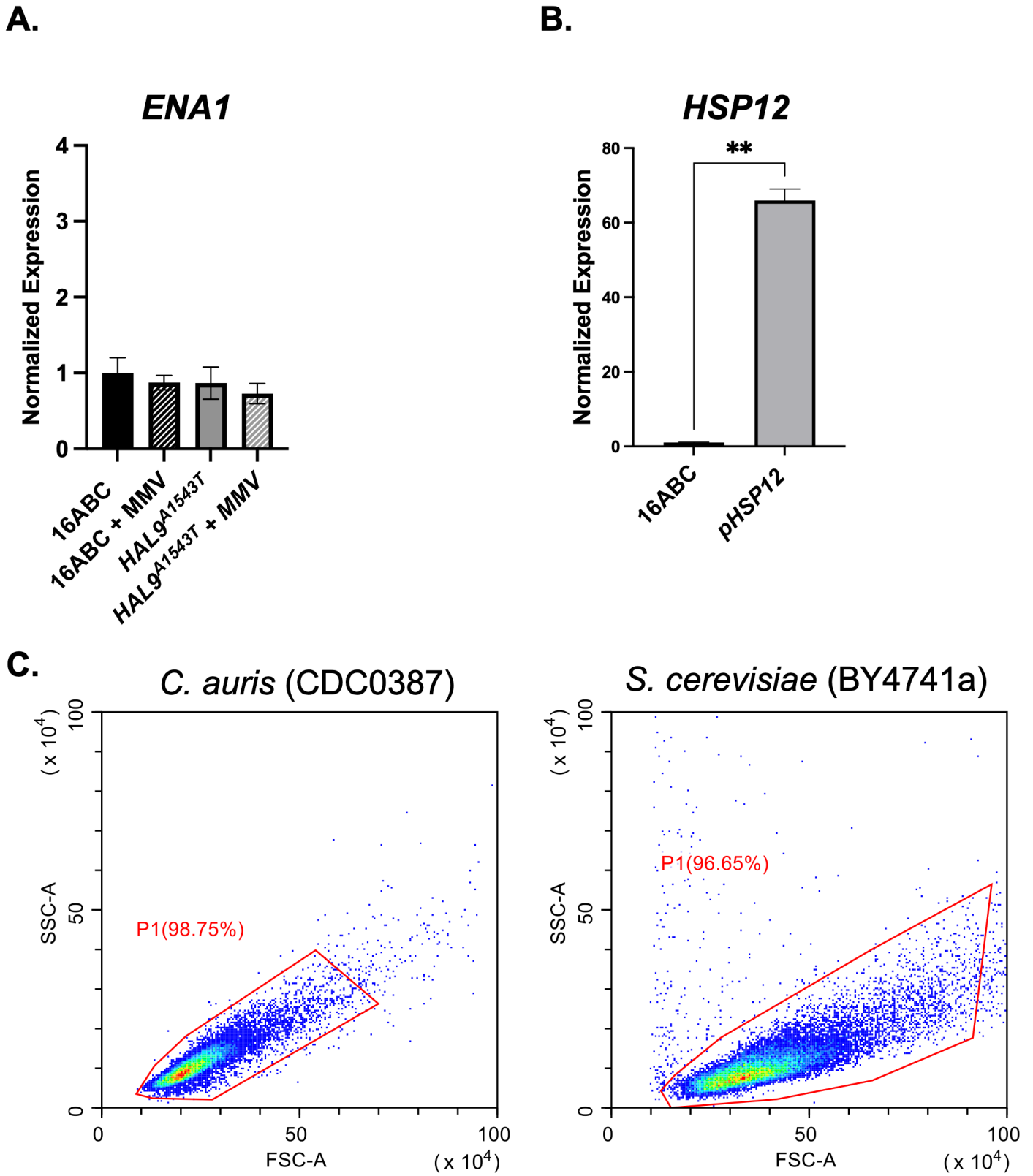

**Figure S5**

Supplement: FIG S5 [file mbio.02730-22-s0005.pdf]
