# Supplementary material for: The Trisubstituted Isoxazole MMV688766 Exerts Broad-Spectrum Activity against Drug-Resistant Fungal Pathogens through Inhibition of Lipid Homeostasis
Source: mBio. 2022 Oct 27;13(6):e02730-22. doi: 10.1128/mbio.02730-22 (PMC9765174; doi:10.1128/mbio.02730-22)
Supplement: FIG S2 [file mbio.02730-22-s0002.pdf]

A.

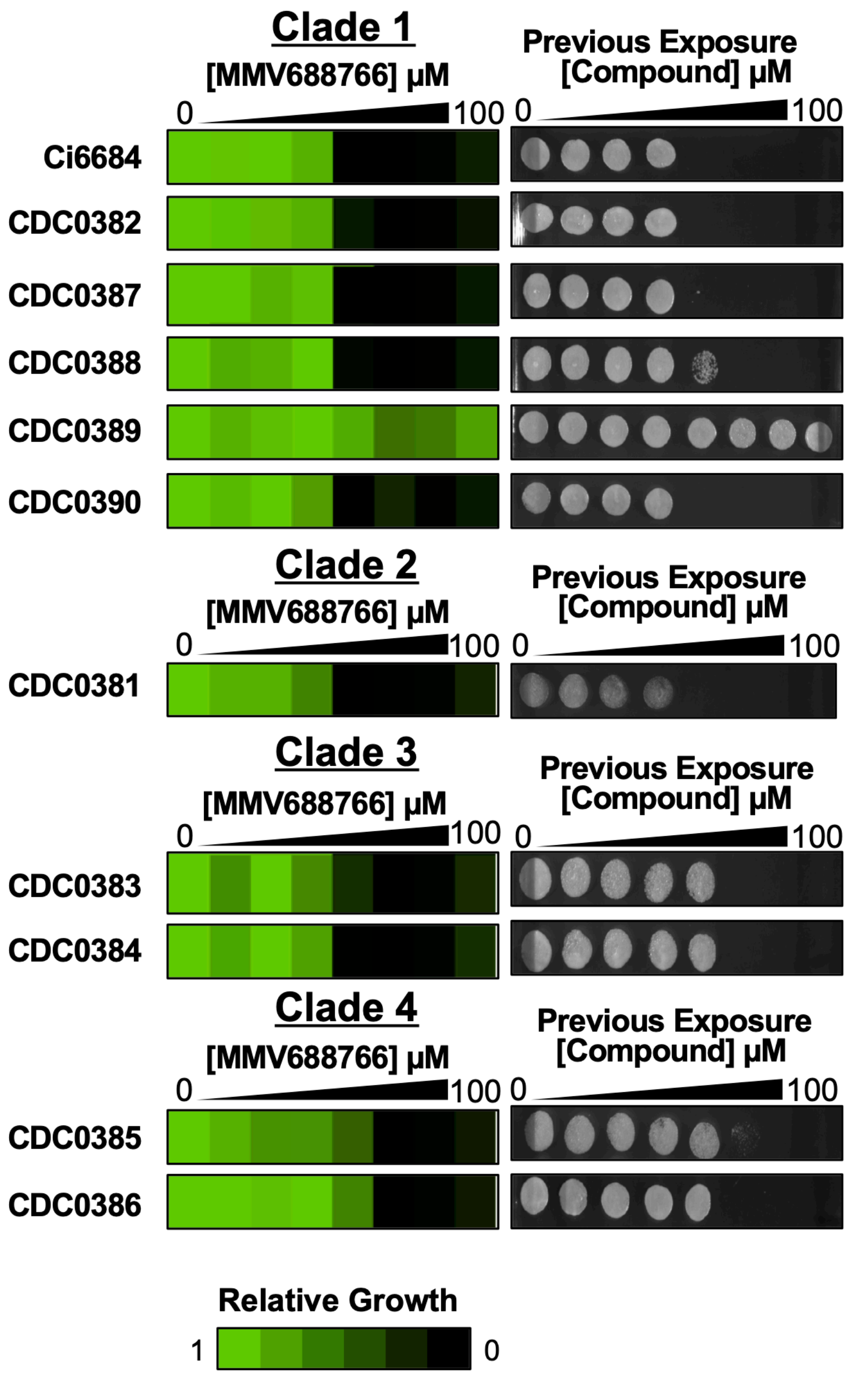

B.

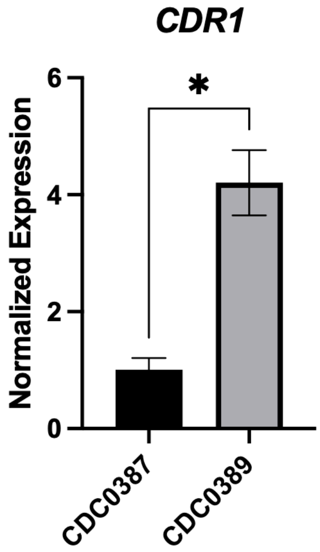

C.

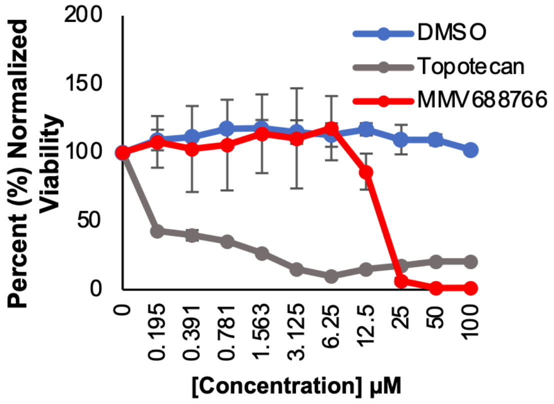

D.

Condition	Treatment	Mean Percent (%) Survival	
		0 hpi	24 hpi
Uninfected	-	100	92
	50 μM MMV688766	100	0
C. albicans infected	-	100	22.67
	50 μM MMV688766	100	0
