# Supplementary material for: The Trisubstituted Isoxazole MMV688766 Exerts Broad-Spectrum Activity against Drug-Resistant Fungal Pathogens through Inhibition of Lipid Homeostasis
Source: mBio. 2022 Oct 27;13(6):e02730-22. doi: 10.1128/mbio.02730-22 (PMC9765174; doi:10.1128/mbio.02730-22)
Supplement: FIG S3 [file mbio.02730-22-s0003.pdf]

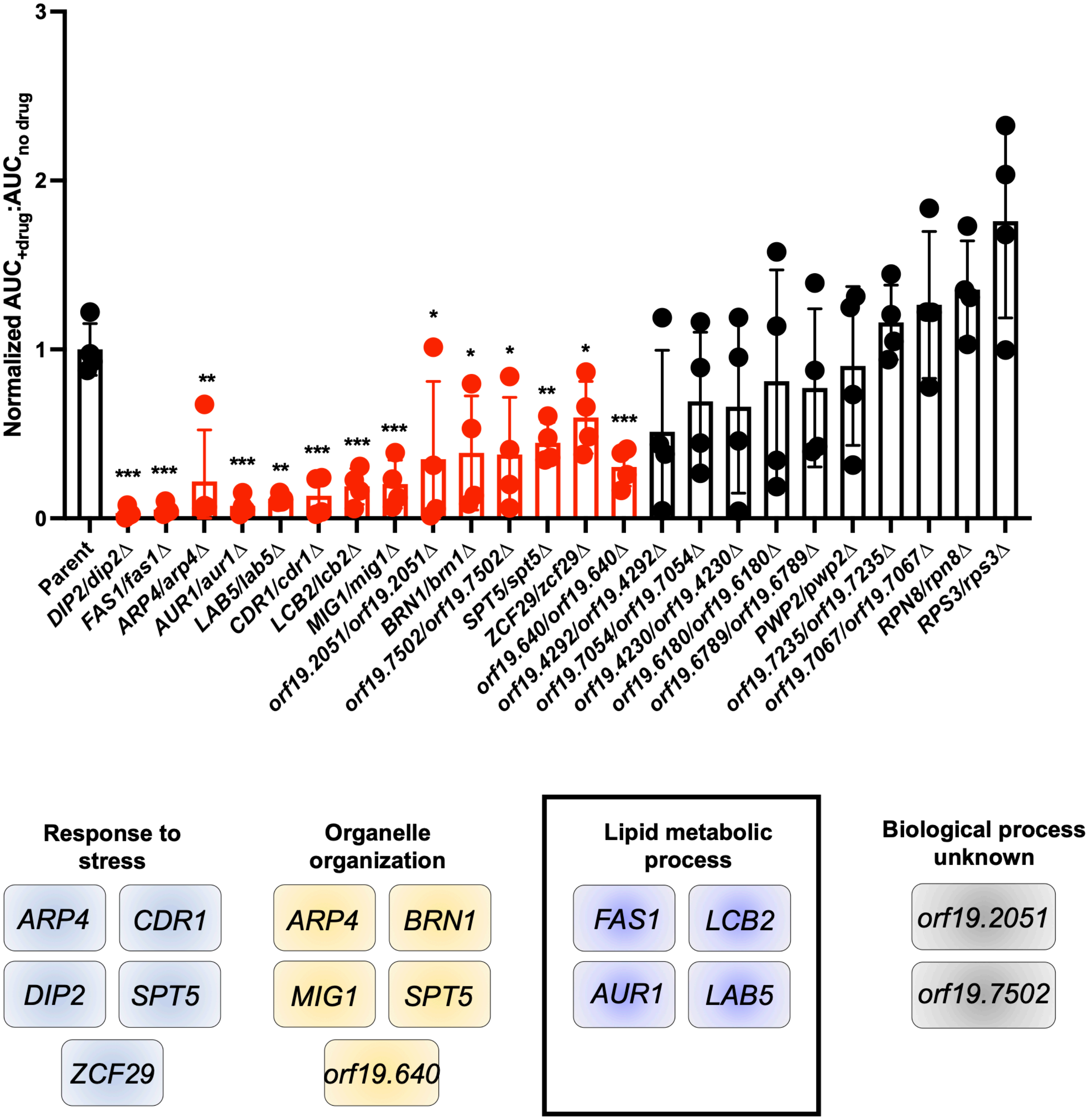

Normalized AUC+drug:AUCno drug
3
2
1
0
Parent
DIP2/dip2Δ
FAS1/fas1Δ
ARP4/arp4Δ
AUR1/aur1Δ
LAB5/lab5Δ
CDR1/cdr1Δ
LCB2/lcb2Δ
MIG1/mig1Δ
orf19.2051/orf19.2051Δ
BRN1/brn1Δ
orf19.7502/orf19.7502Δ
SPT5/spt5Δ
ZCF29/zcf29Δ
orf19.640/orf19.640Δ
orf19.4292/orf19.4292Δ
orf19.7054/orf19.7054Δ
orf19.4230/orf19.4230Δ
orf19.6180/orf19.6180Δ
orf19.6789/orf19.6789Δ
PWP2/pwp2Δ
orf19.7235/orf19.7235Δ
orf19.7067/orf19.7067Δ
RPN8/rpn8Δ
RPS3/rps3Δ
Response to stress
ARP4
CDR1
DIP2
SPT5
ZCF29
Organelle organization
ARP4
BRN1
MIG1
SPT5
orf19.640
Lipid metabolic process
FAS1
LCB2
AUR1
LAB5
Biological process unknown
orf19.2051
orf19.7502
