# Supplementary material for: The Trisubstituted Isoxazole MMV688766 Exerts Broad-Spectrum Activity against Drug-Resistant Fungal Pathogens through Inhibition of Lipid Homeostasis
Source: mBio. 2022 Oct 27;13(6):e02730-22. doi: 10.1128/mbio.02730-22 (PMC9765174; doi:10.1128/mbio.02730-22)
Supplement: FIG S4 [file mbio.02730-22-s0004.pdf]

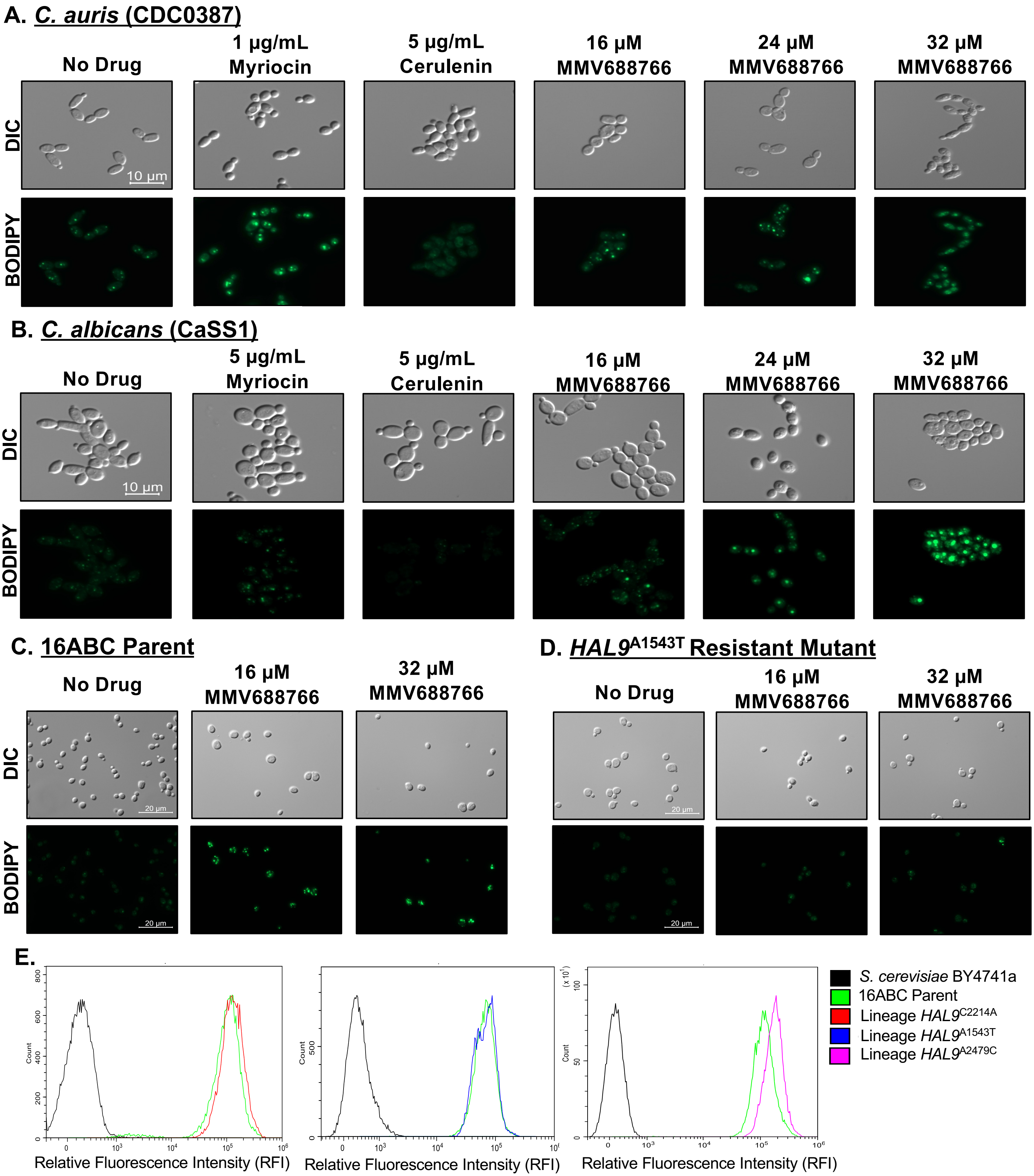

Strain	Events	Mean RFI	Fold-Change RFI
BY4741a	20132	270.63	1
16ABC	20221	98886.77	365.39
Lineage *HAL9*^{C2214A}	20718	122787.0	453.70
Lineage *HAL9*^{A1543T}	21001	66623.5	246.18
Lineage *HAL9*^{A2479C}	18223	172209.7	636.32

Figure S4
