# Supplementary material for: The Trisubstituted Isoxazole MMV688766 Exerts Broad-Spectrum Activity against Drug-Resistant Fungal Pathogens through Inhibition of Lipid Homeostasis
Source: mBio. 2022 Oct 27;13(6):e02730-22. doi: 10.1128/mbio.02730-22 (PMC9765174; doi:10.1128/mbio.02730-22)
Supplement: TEXT S1 [file mbio.02730-22-s0006.pdf]

File S1: Plasmid and strain construction details

Plasmid construction

pLC1543: Plasmid carrying a wild type copy of *HAL9* under the control of a GPD promoter and CYC1 terminator. The gene was amplified from 16ABC (ScLC2336) genomic DNA using oLC9980/oLC9981. The PCR product was spin-column purified and digested using SpeI-HF and BamHI-HF. Plasmid pLC136 was also digested using SpeI-HF and BamHI-HF. Digested products were spin-column purified again and run on agarose gels to ensure correct band size. The purified, digested products were ligated and transformed into DH5alpha and plated on LB with Ampicillin. The presence of the *HAL9* insertion was verified through diagnostic PCR for upstream and downstream integration using oLC1604/oLC9983 and oLC9982/oLC883 respectively.

pLC1544: Plasmid carrying a mutant copy of $HAL9^{C2214A}$ under the control of a GPD promoter and CYC1 terminator. The gene was amplified from ScLC7775 genomic DNA using oLC9980/oLC9981. The PCR product was spin-column purified, and digested using SpeI-HF and BamHI-HF. Plasmid pLC136 was also digested using SpeI-HF and BamHI-HF. Digested products were spin-column purified again, and run on agarose gels to ensure correct band size. The purified, digested products were ligated and transformed into DH5alpha and plated on LB with Ampicillin. The presence of the *HAL9* insertion was verified through diagnostic PCR for upstream and downstream integration using oLC1604/oLC9983 and oLC9982/oLC883 respectively. The *HAL9* SNP C2214A was verified by Sanger sequencing, where the region was amplified using oLC9947/oLC9948, and sequenced using oLC9949.

pLC1545: Plasmid carrying a copy of $HAL9^{A1543T}$ under the control of a GPD promoter and CYC1 terminator. The gene was amplified from ScLC7776 genomic DNA using oLC10k90/oLC10k91. The PCR product was spin-column purified, and digested using BamHI-HF and PstI-HF. Plasmid pLC136 was also digested using BamHI-HF and PstI-HF. Digested products were spin-column purified again, and run on agarose gels to ensure correct band size. The purified, digested products were ligated and transformed into DH5alpha and plated on LB with Ampicillin. The presence of the *HAL9* insertion was verified through diagnostic PCR for upstream and downstream integration using oLC1604/oLC9983 and oLC9982/oLC883 respectively. The *HAL9* SNP A1543T was verified by Sanger sequencing, where the region was amplified using oLC9951/oLC9952, and sequenced using oLC9953.

pLC1546: Plasmid carrying a copy of $HAL9^{A2479C}$ under the control of a GPD promoter and CYC1 terminator. The gene was amplified from ScLC7777 genomic DNA using oLC9980/oLC9981. The PCR product was spin-column purified, and digested using SpeI-HF and BamHI-HF. Plasmid pLC136 was also digested using SpeI-HF and BamHI-HF. Digested products were spin-column purified again, and run on agarose gels to ensure correct band size. The purified, digested products were ligated and transformed into DH5alpha and plated on LB with Ampicillin. The presence of the *HAL9* insertion was verified through diagnostic PCR for upstream and downstream integration using oLC1604/oLC9983 and oLC9982/oLC883 respectively. The *HAL9* SNP A2479C was verified by Sanger sequencing, where the region was amplified using oLC9955/oLC9956, and sequenced using oLC9958.

pLC1609: Plasmid carrying a WT copy of *HSP12* under the control of a GPD promoter and CYC1 terminator. The gene was amplified from ScLC151 genomic DNA using oLC10k491/oLC10k492. The PCR product was spin-column purified, and digested using BamHI-HF and SalI-HF. Plasmid pLC137 was also digested using BamHI-HF and SalI-HF. Digested products were spin-column purified again, and run on agarose gels to ensure correct band size. The purified, digested products were ligated (T4 ligase), transformed into DH5alpha and plated on LB with Ampicillin. The presence of the *HSP12* insertion was verified through diagnostic PCR for upstream and downstream integration using oLC805/oLC10k493 and oLC10k494/oLC883 respectively.

Strain construction

ScLC7775: 16ABC (ScLC2336) was subject to liquid selection via serial passaging to a final concentration of 150 µM MMV688766 in YPD (shaking). This mutant was isolated from lineage B, clone 5.5. *HAL9*^{C2214A} mutation was identified through WGS and verified through sanger sequencing using oLC9947/oLC9948 to amplify to amplify relevant region of *HAL9* and oLC9949 or oLC9950 to sequence.

ScLC7776: 16ABC (ScLC2336) was subject to liquid selection via serial passaging to a final concentration of 150 µM MMV688766 in YPD (shaking). This mutant was isolated from lineage C, clone 1.6. Mutation *HAL9*^{A1543} identified through WGS and verified through sanger sequencing using oLC9951/oLC9952 to amplify relevant region of *HAL9* and oLC9953 or oLC9954 to sequence.

ScLC7777: 16ABC (ScLC2336) was subject to liquid selection via serial passaging to a final concentration of 150 µM MMV688766 in YPD (shaking). This mutant was isolated from lineage D, clone 3.3. Mutation *HAL9*^{A2479C} identified through WGS and verified through sanger sequencing using oLC9955/oLC9956 to amplify relevant region of *HAL9* and oLC9957 or oLC9958 to sequence.

ScLC7861: The HygB resistance cassette was amplified from pLC3 using oLC9974/oLC9975, each containing 70bp homology to the 5' and 3' of the *HAL9* orf. ScLC151 (BY4741) was transformed (LiAc) with the PCR amplified HygB cassette and plated on YPD with 200 µg/mL HygB. Integration of HygB at the *HAL9* locus was verified with primer pairs oLC10k69/oLC59 (upstream) and oLC60/oLC10k70 (downstream).

ScLC7862: The HygB resistance cassette was amplified from ScLC7861 using oLC10k69/oLC10k70 to include ~250 bp homology to the 5' and 3' of the *HAL9* orf. ScLC2336 (16ABC) was transformed (LiAc) with the PCR amplified HygB cassette and plated on YPD with 200 µg/mL HygB. Integration of HygB at the *HAL9* locus was verified with primer pairs oLC10k165/oLC59 (upstream) and oLC60/oLC10k166 (downstream).

ScLC7863: The HygB resistance cassette was amplified from ScLC7861 using oLC10k69/oLC10k70 to include ~250 bp homology to the 5' and 3' of the *HAL9* orf. ScLC7776 (*HAL9*^{A1543T}) was transformed (LiAc) with the PCR amplified HygB cassette and plated on YPD with 200 µg/mL HygB. Integration of HygB at the *HAL9* locus was verified with primer pairs oLC10k165/oLC59 (upstream) and oLC60/oLC10k166 (downstream).

ScLC7864: The HygB resistance cassette was amplified from ScLC7861 using oLC10k69/oLC10k70 to include ~250 bp homology to the 5' and 3' of the *HAL9* orf. ScLC7777 ($HAL9^{A2479C}$) was transformed (LiAc) with the PCR amplified HygB cassette and plated on YPD with 200 µg/mL HygB. Integration of HygB at the *HAL9* locus was verified with primer pairs oLC10k165/oLC59 (upstream) and oLC60/oLC10k166 (downstream).

ScLC7937: ScLC7862 was transformed with pLC1543 using standard LiOAc yeast transformation protocol. Transformants were selected on SD plates supplemented with Met (selecting for Leu prototrophs). Colonies were patched on the same plates and PCR tested with oLC1604/oLC9983 (upstream) and oLC9982/oLC883 (downstream) to confirm the presence of the plasmid, as well as integration of *HAL9* in the plasmid. Integration of HygB at the *HAL9* locus was also verified with primer pairs oLC10k165/oLC59 (upstream) and oLC60/oLC10k166 (downstream).

ScLC7938: ScLC7862 was transformed with pLC1544 using standard LiOAc yeast transformation protocol. Transformants were selected on SD plates supplemented with Met (selecting for Leu prototrophs). Colonies were patched on the same plates and PCR tested with oLC1604/oLC9983 (upstream) and oLC9982/oLC883 (downstream) to confirm the presence of the plasmid, as well as integration of *HAL9* in the plasmid. Integration of HygB at the *HAL9* locus was also verified with primer pairs oLC10k165/oLC59 (upstream) and oLC60/oLC10k166 (downstream).

ScLC7939: ScLC7862 was transformed with pLC1545 using standard LiOAc yeast transformation protocol. Transformants were selected on SD plates supplemented with Met (selecting for Leu prototrophs). Colonies were patched on the same plates and PCR tested with oLC1604/oLC9983 (upstream) and oLC9982/oLC883 (downstream) to confirm the presence of the plasmid, as well as integration of *HAL9* in the plasmid. Integration of HygB at the *HAL9* locus was also verified with primer pairs oLC10k165/oLC59 (upstream) and oLC60/oLC10k166 (downstream).

ScLC7940: ScLC7862 was transformed with pLC1546 using standard LiOAc yeast transformation protocol. Transformants were selected on SD plates supplemented with Met (selecting for Leu prototrophs). Colonies were patched on the same plates and PCR tested with oLC1604/oLC9983 (upstream) and oLC9982/oLC883 (downstream) to confirm the presence of the plasmid, as well as integration of *HAL9* in the plasmid. Integration of HygB at the *HAL9* locus was also verified with primer pairs oLC10k165/oLC59 (upstream) and oLC60/oLC10k166 (downstream).

ScLC7941: ScLC7863 was transformed with pLC1543 using standard LiOAc yeast transformation protocol. Transformants were selected on SD plates supplemented with Met (selecting for Leu prototrophs). Colonies were patched on the same plates and PCR tested with oLC1604/oLC9983 (upstream) and oLC9982/oLC883 (downstream) to confirm the presence of the plasmid, as well as integration of *HAL9* in the plasmid. Integration of HygB at the *HAL9* locus was also verified with primer pairs oLC10k165/oLC59 (upstream) and oLC60/oLC10k166 (downstream).

ScLC7942: ScLC7863 was transformed with pLC1544 using standard LiOAc yeast transformation protocol. Transformants were selected on SD plates supplemented with Met (selecting for Leu prototrophs). Colonies were patched on the same plates and PCR tested with oLC1604/oLC9983 (upstream) and oLC9982/oLC883 (downstream) to confirm the presence of the plasmid, as well as integration of *HAL9* in the plasmid. Integration of HygB at the *HAL9* locus was also verified with primer pairs oLC10k165/oLC59 (upstream) and oLC60/oLC10k166 (downstream).

ScLC7943: ScLC7863 was transformed with pLC1545 using standard LiOAc yeast transformation protocol. Transformants were selected on SD plates supplemented with Met (selecting for Leu prototrophs). Colonies were patched on the same plates and PCR tested with oLC1604/oLC9983 (upstream) and oLC9982/oLC883 (downstream) to confirm the presence of the plasmid, as well as integration of *HAL9* in the plasmid. Integration of HygB at the *HAL9* locus was also verified with primer pairs oLC10k165/oLC59 (upstream) and oLC60/oLC10k166 (downstream).

ScLC7944: ScLC7863 was transformed with pLC1546 using standard LiOAc yeast transformation protocol. Transformants were selected on SD plates supplemented with Met (selecting for Leu prototrophs). Colonies were patched on the same plates and PCR tested with oLC1604/oLC9983 (upstream) and oLC9982/oLC883 (downstream) to confirm the presence of the plasmid, as well as integration of *HAL9* in the plasmid. Integration of HygB at the *HAL9* locus was also verified with primer pairs oLC10k165/oLC59 (upstream) and oLC60/oLC10k166 (downstream).

ScLC8331: The HygB resistance cassette was amplified from pLC3 using oLC10k459/oLC10k460 each containing 70bp homology to the 5' and 3' of the *HSP12* orf. ScLC151 (BY4741a) was transformed (LiAc) with the PCR amplified HygB cassette and plated on YPD with 100 µg/mL HygB. Integration of HygB at the *HSP12* locus was verified with primer pairs oLC10k471/oLC59 (upstream), oLC60/oLC10k472 (downstream), and oLC10k352/oLC10k353 (internal).

ScLC8332: The LEU2 cassette was amplified from pLC43 using oLC10k457/oLC10k458, each containing 70bp homology to the 5' and 3' of the *MSN2* orf. ScLC151 (BY4741) was transformed (LiAc) with the PCR amplified LEU2 cassette and plated on SD (+His+Met+Ura-Leu). Integration of *LEU2* at the *MSN2* locus was verified with primer pairs oLC10k469/oLC386 (upstream), oLC387/oLC10k470 (downstream), and oLC10k501/oLC10k502 (internal).

ScLC8333: The HygB resistance cassette was amplified from pLC3 using oLC10k455/ oLC10k456, each containing 70bp homology to the 5' and 3' of the *MSN4* orf. ScLC151 (BY4741) was transformed (LiAc) with the PCR amplified HygB cassette and plated on YPD with 100 µg/mL HygB. Integration of HygB at the *MSN4* locus was verified with primer pairs oLC10k467/oLC59 (upstream), oLC60/oLC10k468 (downstream), and oLC10k340/oLC10k341 (internal).

ScLC8334: The HygB resistance cassette was amplified from ScLC8331 using oLC10k465/oLC10k466 to include ~300 bp homology to the 5' and 3' of the *HSP12* orf. ScLC2336 (GMa) was transformed (LiAc) with the PCR amplified HygB cassette and plated on YPD with 100 µg/mL HygB. Integration of HygB at the *HSP12* locus was verified with primer pairs oLC10k495/oLC59 (upstream), oLC60/oLC10k496 (downstream), oLC10k352/oLC10k353 (internal).

ScLC8335: The HygB resistance cassette was amplified from ScLC8331 using oLC10k465/oLC10k466 to include ~300 bp homology to the 5' and 3' of the *HSP12* orf. ScLC2336 (GMa) was transformed (LiAc) with the PCR amplified HygB cassette and plated on YPD with 100 µg/mL HygB. Integration of HygB at the *HSP12* locus was verified with primer pairs oLC10k495/oLC59 (upstream), oLC60/oLC10k496 (downstream), oLC10k352/oLC10k353 (internal).

ScLC8336: The LEU2 cassette was amplified from ScLC8332 using oLC10k463/oLC10k464 to include ~300 bp homology to the 5' and 3' of the *HSP12* orf. ScLC2336 (GMa) was transformed (LiAc) with the PCR amplified HygB cassette and plated on SD with Met. Integration of HygB at the *HSP12* locus was verified with primer pairs oLC10k497/oLC386 (upstream), oLC387/oLC10k498 (downstream), oLC10k501/oLC10k502 (internal).

ScLC8337: The LEU2 cassette was amplified from ScLC8332 using oLC10k463/oLC10k464 to include ~300 bp homology to the 5' and 3' of the *HSP12* orf. ScLC2336 (GMa) was transformed (LiAc) with the PCR amplified HygB cassette and plated on SD with Met. Integration of HygB at the *HSP12* locus was verified with primer pairs oLC10k497/oLC386 (upstream), oLC387/oLC10k498 (downstream), oLC10k501/oLC10k502 (internal).

ScLC8338: The HygB resistance cassette was amplified from ScLC8333 using oLC10k461/oLC10k462 to include ~300 bp homology to the 5' and 3' of the *MSN4* orf. ScLC2336 (GMa) was transformed (LiAc) with the PCR amplified HygB cassette and plated on YPD with 100 µg/mL HygB. Integration of HygB at the *MSN4* locus was verified with primer pairs oLC10k499/oLC59 (upstream), oLC60/oLC10k500 (downstream), oLC10k340/oLC10k341 (internal).

ScLC8339: The HygB resistance cassette was amplified from ScLC8333 using oLC10k461/oLC10k462 to include ~300 bp homology to the 5' and 3' of the *MSN4* orf. ScLC7776 was transformed (LiAc) with the PCR amplified HygB cassette and plated on YPD+100 µg/mL HygB. Integration of HygB at the *MSN4* locus was verified with primer pairs oLC10k499/oLC59 (upstream), oLC60/oLC10k500 (downstream), oLC10k340/oLC10k341 (internal).

ScLC8340: The 16ABC confirmed *MSN2::LEU2* strain ScLC8336 underwent a second transformation to further delete *MSN4.* HygB resistance cassette was amplified from ScLC8336 using oLC10k461/oLC10k462 to include ~300 bp homology to the 5' and 3' of the *MSN4* orf. ScLC2336 (16ABC) was transformed (LiAc) with the PCR amplified HygB cassette and plated on YPD+100 µg/mL HygB. Integration of HygB at the *MSN4* locus, as well as confirmation of integration of *LEU2* at the *MSN2* locus was verified with primer pairs oLC10k499/oLC59

(*MSN4*::HygB upstream), oLC60/oLC10k500 (*MSN4*::HygB downstream), oLC10k340/oLC10k341 (*MSN4* orf), and oLC10k497/oLC386 (*MSN2::LEU2* upstream), oLC387/oLC10k498 (*MSN2::LEU2* downstream), oLC10k501/oLC10k502 (*MSN2* internal).

ScLC8341: The confirmed *MSN2::LEU2* strain ScLC8337 (ScLC7776 *MSN2::LEU2*) underwent a second transformation to further delete *MSN4*. HygB resistance cassette was amplified from ScLC8337 using oLC10k461/oLC10k462 to include ~300 bp homology to the 5' and 3' of the *MSN4* orf. ScLC7776 was transformed (LiAc) with the PCR amplified HygB cassette and plated on YPD+100 µg/mL HygB. Integration of HygB at the *MSN4* locus, as well as confirmation of integration of *LEU2* at the *MSN2* locus was verified with primer pairs oLC10k499/oLC59 (*MSN4*::HygB upstream), oLC60/oLC10k500 (*MSN4*::HygB downstream), oLC10k340/oLC10k341 (*MSN4* orf), and oLC10k497/oLC386 (*MSN2::LEU2* upstream), oLC387/oLC10k498 (*MSN2::LEU2* downstream), oLC10k501/oLC10k502 (*MSN2* internal).

ScLC8342: ScLC2336 (16ABC) was transformed with pLC1609 using standard LiAc yeast transformation protocol. Transformants were selected on SD plates supplemented with Met (selecting for Leu prototrophs). Colonies were patched on the same plates and PCR tested with oLC805/oLC10k493 (upstream), oLC10k494/oLC883 (downstream), oLC805/oLC883 (GPD-CYC1) to confirm the presence of the plasmid, as well as integration of *HSP12* in the plasmid.
