# Supplementary material for: The Trisubstituted Isoxazole MMV688766 Exerts Broad-Spectrum Activity against Drug-Resistant Fungal Pathogens through Inhibition of Lipid Homeostasis
Source: mBio. 2022 Oct 27;13(6):e02730-22. doi: 10.1128/mbio.02730-22 (PMC9765174; doi:10.1128/mbio.02730-22)
Supplement: TABLE S1 [file mbio.02730-22-s0007.pdf]

Table S1: Strains used in this study

Strain name	Strain ID	Species	Genotype	Source
Ci6684	CauLC5083	*C. auris*	Clinical isolate	[1]
CDC0381 (B11220)	CauLC5288	*C. auris*	CDC panel clinical isolate	[2,3]
CDC0382 (B11109)	CauLC5289	*C. auris*	CDC panel clinical isolate	[2,3]
CDC0383 (B1121)	CauLC5290	*C. auris*	CDC panel clinical isolate	[2,3]
CDC0384 (B11222)	CauLC5291	*C. auris*	CDC panel clinical isolate	[2,3]
CDC0385 (B11244)	CauLC5292	*C. auris*	CDC panel clinical isolate	[2,3]
CDC0386 (B11245)	CauLC5293	*C. auris*	CDC panel clinical isolate	[2,3]
CDC0387 (B8441)	CauLC5294	*C. auris*	CDC panel clinical isolate	[2,3]
CDC0388 (B11098)	CauLC5295	*C. auris*	CDC panel clinical isolate	[2,3]
CDC0389 (B11203)	CauLC5296	*C. auris*	CDC panel clinical isolate	[2,3]
CDC0390 (B11205)	CauLC5297	*C. auris*	CDC panel clinical isolate	[2,3]
CaSS1	CaLC6106	*C. albicans*	ura3::imm434/ura3::imm434 his3::hisG/his3::hisG leu2::tetRGAL4AD-URA/LEU2	[4,5]
SN95	CaLC239	*C. albicans*	*arg4Δ/arg4Δ his1Δ/his1Δ URA3/ura3Δ::imm434 IRO1/iro1Δ::imm434*	[6]
BG2	CgLC1002	*C. glabrata*	Wild type *C. glabrata*	[7]
H99	CnLC3142	*C. neoformans*	Wild type *C. neoformans*	[8]
Af239	AfLC3669	*A. fumigatus*	Wild type *A. fumigatus*	[9]
BJ2168 3xFLAG-*FAS1*	JWL01	*S. cerevisiae*	*leu2Δ trp1Δ ura3–52 prb1-1122 pep4-3 prc1-407 gal2Δ FAS1*-3xFLAG	[10]
SN95 6HIS3xFLAG-*FAS1*	CaLC5425	*C. albicans*	arg4Δ/arg4Δ *his1Δ/his1Δ FAS1-6HIS3xFLAG HIS1/fas1Δ*	[10]
BY4741a	ScLC151	*S. cerevisiae*	*his3Δ leu2Δ met15Δ ura3Δ*	[11]
16ABC	ScLC2336	*S. cerevisiae*	*adp1Δ snq2Δ ycf1Δ pdr15Δ yor1Δ vmr1Δ pdr11Δ nft1Δ bpt1Δ ybt1Δ ynr070wΔ yol075cΔ aus1Δ pdr5Δ pdr10Δ pdr12Δ can1Δ::GMToolkit*-a *his3Δ1 leu2Δ0 ura3Δ0 met15Δ0*	[12]
16ABC *HAL9*^{C2214A}	ScLC7775	*S. cerevisiae*	As ScLC2336, *HAL9*^{C2214A}	This study
16ABC *HAL9*^{A1543T}	ScLC7776	*S. cerevisiae*	As ScLC2336, *HAL9*^{A1543T}	This study
16ABC *HAL9*^{A2479T}	ScLC7777	*S. cerevisiae*	As ScLC2336, *HAL9*^{A2479T}	This study
BY4741a *hal9Δ*	ScLC7861	*S. cerevisiae*	As ScLC151, *HAL9*::HYGB	This study

16ABC *hal9*Δ	ScLC7862	*S. cerevisiae*	As ScLC2336, *HAL9*::HYGB	This study
16ABC *hal9*^{A1543T}Δ	ScLC7863	*S. cerevisiae*	As ScLC2336, *HAL9*^{A1543T}::HYGB	This study
16ABC *hal9*Δ + *pHAL9*	ScLC7937	*S. cerevisiae*	As ScLC7862 + pLC1543 (*LEU2*)	This study
16ABC *hal9*Δ + *pHAL9*^{C2214A}	ScLC7938	*S. cerevisiae*	As ScLC7862 + pLC1544 (*LEU2*)	This study
16ABC *hal9*Δ + *pHAL9*^{A1543T}	ScLC7939	*S. cerevisiae*	As ScLC7862 + pLC1545 (*LEU2*)	This study
16ABC *hal9*Δ + *HAL9*^{A2479T}	ScLC7940	*S. cerevisiae*	As ScLC7862 + pLC1546 (*LEU2*)	This study
16ABC *hal9*^{A1543T}Δ + *pHAL9*	ScLC7941	*S. cerevisiae*	As ScLC7863 + pLC1543 (*LEU2*)	This study
16ABC *hal9*^{A1543T}Δ + *pHAL9*^{C2214A}	ScLC7942	*S. cerevisiae*	As ScLC7863 + pLC1544 (*LEU2*)	This study
16ABC *hal9*^{A1543T}Δ + *pHAL9*^{A1543T}	ScLC7943	*S. cerevisiae*	As ScLC7863 + pLC1545 (*LEU2*)	This study
16ABC *hal9*^{A1543T}Δ+ *HAL9*^{A2479T}	ScLC7944	*S. cerevisiae*	As ScLC7863 + pLC1546 (*LEU2*)	This study
BY4741a *HSP12*::HygB	ScLC8331	*S. cerevisiae*	As ScLC151, *HSP12*::HYGB	This study
BY4741a *MSN2*::*LEU2*	ScLC8332	*S. cerevisiae*	As ScLC151, *MSN2::LEU2*	This study
BY4741a *MSN4*::HygB	ScLC8333	*S. cerevisiae*	As ScLC151, *MSN4*::HYGB	This study
16ABC *hsp12*Δ	ScLC8334	*S. cerevisiae*	As ScLC2336, *HSP12::HygB*	This study
HAL9^{A1543T} *hsp12*Δ	ScLC8335	*S. cerevisiae*	As ScLC7776, *HSP12::HygB*	This study
16ABC *msn2*Δ	ScLC8336	*S. cerevisiae*	As ScLC2336, *MSN2::LEU2*	This study
HAL9^{A1543T} *msn2*Δ	ScLC8337	*S. cerevisiae*	As ScLC2336, *MSN2::LEU2*	This study
16ABC *msn4*Δ	ScLC8338	*S. cerevisiae*	As ScLC2336, *MSN4::HygB*	This study
HAL9^{A1543T} *msn4*Δ	ScLC8339	*S. cerevisiae*	As ScLC2336, *MSN4::HygB*	This study
16ABC *msn2*Δ*msn4*Δ	ScLC8340	*S. cerevisiae*	As ScLC2336, *MSN2::LEU2 MSN4::HygB*	This study
HAL9^{A1543T} *msn2*Δ*msn4*Δ	ScLC8341	*S. cerevisiae*	As ScLC7776, *MSN2::LEU2 MSN4::HygB*	This study
16ABC p*HSP12*^{OE}	ScLC8341	*S. cerevisiae*	As ScLC2336 + pLC1609 (*LEU2*)	This study

References

1. Chatterjee S, Alampalli SV, Nageshan RK, Chettiar ST, Joshi S, Tatu US. Draft genome of a commonly misdiagnosed multidrug resistant pathogen *Candida auris*. *BMC Genomics*. 2015;16(1):1-16. doi:10.1186/s12864-015-1863-z
2. Lockhart SR, Etienne KA, Vallabhaneni S, et al. Simultaneous emergence of multidrug-resistant *Candida auris* on 3 continents confirmed by whole-genome sequencing and epidemiological analyses. *Clin Infect Dis*. 2017;64(2):134-140. doi:10.1093/cid/ciw691
3. CDC & FDA Antibiotic Resistance Isolate Bank. Atlanta (GA):CDC (2020).
4. Roemer T, Jiang B, Davison J, et al. Large-scale essential gene identification in *Candida albicans* and applications to antifungal drug discovery. *Mol Microbiol*. 2003;50(1):167-181. doi:10.1046/j.1365-2958.2003.03697.x
5. Fu C, Zhang X, Veri AO, et al. Leveraging machine learning essentiality predictions and chemogenomic interactions to identify antifungal targets. *Nat Commun*. 2021;12(1):1-18. doi:10.1038/s41467-021-26850-3
6. Noble SM, Johnson AD. Strains and strategies for large-scale gene deletion studies of the diploid human fungal pathogen *Candida albicans*. *Eukaryot Cell*. 2005;4(2):298-309. doi:10.1128/EC.4.2.298-309.2005
7. Fidel PL, Cutright JL, Tait L, Sobel JD. A murine model of *Candida glabrata* vaginitis. *J Infect Dis*. 1996;173(2):425-431. doi:10.1093/infdis/173.2.425
8. Granger DL, Perfect JR, Durack DT. Virulence of *Cryptococcus neoformans:* Regulation of capsule synthesis by carbon dioxide. *J Clin Invest*. 1985;76(2):508-516. doi:10.1172/JCI112000
9. Nierman WC, Pain A, Anderson MJ, et al. Genomic sequence of the pathogenic and allergenic filamentous fungus *Aspergillus fumigatus*. *Nature*. 2005;438(7071):1151-1156. doi:10.1038/nature04332
10. Lou JW, Iyer KR, Hasan SMN, Cowen LE, Mazhab-Jafari MT. Electron cryomicroscopy observation of acyl carrier protein translocation in type I fungal fatty acid synthase. *Sci Rep*. 2019;9(1):1-8. doi:10.1038/s41598-019-49261-3
11. Giaever G, Chu AM, Ni L, et al. Functional profiling of the *Saccharomyces cerevisiae* genome. *Nature*. 2002;418(6896):387-391. doi:10.1038/nature00935
12. Suzuki Y, Onge RPS, Mani R, et al. Knocking out multigene redundancies via cycles of sexual assortment and fluorescence selection. *Nat Methods*. 2011;8(2):159-164. doi:10.1038/nmeth.1550
