# Supplementary material for: The Trisubstituted Isoxazole MMV688766 Exerts Broad-Spectrum Activity against Drug-Resistant Fungal Pathogens through Inhibition of Lipid Homeostasis
Source: mBio. 2022 Oct 27;13(6):e02730-22. doi: 10.1128/mbio.02730-22 (PMC9765174; doi:10.1128/mbio.02730-22)
Supplement: TABLE S2 [file mbio.02730-22-s0008.pdf]

Table S2: Plasmids used in this study

Plasmid number	Description	Source
pLC3	ampR, HYGB	[1,2]
pLC136	ampR, *GPD1p*, *CYC1t*, *LEU2* (p415GPD)	[3]
pLC1543	*GPD1p-HAL9-CYCt* (ampR, *LEU2*)	This study
pLC1544	*GPD1p-HAL9*^{C2214A}-*CYCt* (ampR, *LEU2*)	This study
pLC1545	*GPD1p-HAL9*^{A1543T}-*CYCt* (ampR, *LEU2*)	This study
pLC1546	*GPD1p-HAL9*^{A2479C} -*CYCt* (ampR, *LEU2*)	This study
pLC1609	*GPD1p-HSP12-CYCt* (ampR, *LEU2*)	This study

References

1. Wach A, Brachat A, Pöhlmann R, Philippsen P. New heterologous modules for classical or PCR-based gene disruptions in *Saccharomyces cerevisiae. Yeast*. 1994;10(13):1793-1808. doi:10.1002/yea.320101310
2. Goldstein AL, McCusker JH. Three new dominant drug resistance cassettes for gene disruption in *Saccharomyces cerevisiae. Yeast*. 1999;15(14):1541-1553. doi:10.1002/(SICI)1097-0061(199910)15:14<1541::AID-YEA476>3.0.CO;2-K
3. Mumberg D, Müller R, Funk M. Yeast vectors for the controlled expression of heterologous proteins in different genetic backgrounds. *Gene*. 1995;156(1):119-122. doi:https://doi.org/10.1016/0378-1119(95)00037-7
