# Supplementary material for: The Trisubstituted Isoxazole MMV688766 Exerts Broad-Spectrum Activity against Drug-Resistant Fungal Pathogens through Inhibition of Lipid Homeostasis
Source: mBio. 2022 Oct 27;13(6):e02730-22. doi: 10.1128/mbio.02730-22 (PMC9765174; doi:10.1128/mbio.02730-22)
Supplement: TABLE S3 [file mbio.02730-22-s0009.pdf]

Table S3: Oligonucleotides used in this study

Primer name	Description	Sequence
UP-TAG universal amplification primer	For HIP	AATGATACGGCGACCACCGAGATCTACACCGAGGTCGAGAATGATGTCCACGAGGTCTCT
DOWN-TAG universal amplification primer	For HIP	AATGATACGGCGACCACCGAGATCTACACCACATGATATGTTGAGCGGTGTCGGTCTCGTAG
UP-TAG index amplification primer[1]	For HIP	CAAGCAGAAGACGGCATACGAGATNNNNNNGCCATTTGTCTGTCGACCTGCAGCGTACG
DOWN-TAG index amplification primer[1]	For HIP	CAAGCAGAAGACGGCATACGAGATNNNNNNGAGTATCTGTATCTGGCCGAGCTCGAATTCATCGA
UP-TAG sequencing primer	For HIP	CGAGGTCGAGAATGATGTCCACGAGGTCTCT
DOWN-TAG sequencing primer	For HIP	CACATGATATGTTGAGCGGTGTCGGTCTCGTAG
UP-TAG index sequencing primer	For HIP	CGTACGCTGCAGGTCGACAGACAAATGGC
DOWN-TAG index sequencing primer	For HIP	ATCGATGAATTCGAGCTCGGCCAGATACAGATACTC
oLC9947	*ScHAL9*_orf_1947_C2214A-Amp-Fwd	CAATTGGAGAGACAACTTGC
oLC9948	*ScHAL9*_orf_2451_C2214A-Amp-Rev	GACATTGTCAAACAGTAACTGC
oLC9951	*ScHAL9*_orf_1261_A1543T-Amp-Fwd	GCCACTCAATCAATTATAAGAGG
oLC9952	*ScHAL9*_orf_1912_A1543T-Amp-Rev	GCATGGCATCGAATGAAAG
oLC9955	*ScHAL9*_orf_2242_A2479T-Amp-Fwd	CTGTTTCAAGGCATCAATTACC
oLC9956	*ScHAL9*_orf_2751_A2479T-Amp-Rev	GTTTATTGGTGCTTCCTCCG
oLC9949	*ScHAL9*_orf_2007_C2214A-Seq-Fwd	GGTACTTTACGCTCAAAAATCC
oLC9950	*ScHAL9*_orf_2383_C2214A- Seq -Rev	CGATGATCTCTTTTACCTCACC
oLC9953	*ScHAL9*_orf_1350_A1543T-Seq-Fwd	CGTTGCCTTACTAAATGCCA
oLC9954	*ScHAL9*_orf_1783_A1543T-Seq-Rev	CAACTACATTCAATGCAGAGTTG
oLC9957	*ScHAL9*_orf_2343_A2479T-Seq-Fwd	GAACGATCTAAAAAAGAAAGGTGAGG
oLC9958	*ScHAL9*_orf_2668_A2479T-Seq-Rev	CCTTTTGTAAAGAATGGAGAGAACC
oLC9974	HygB+70bp 5' UTR *ScHAL9*	AGCTTATTATCGTACTTTAATTCAACTTAGGAAATCAGCACTCTCCACTCCAAATCTTCGGTTTGAAATAACATGGAGGCCCAGAATACC

oLC9975	HygB+70bp 3' UTR *ScHAL9*	CAAATAGTACATATAATATGCATTGGGAAAGAATTTTTATTTTTTACAATCTTTGTAGACAAGGTACAGTCAGTATAGCGACCAGCATTC
oLC10k69	*ScHAL9*-245bp_Fwd	AAGGTCTACCACATGTGTTTTGAAT
oLC10k70	*ScHAL9*+255bp_Rev	GTTTACATGACCCAAGCAAAATTAC
oLC9980	Sc*HAL9*+1Fwd-SpeI	CGCTACTAGTATGGAAAATCAA GGTGGAGATTACAGC
oLC9981	*ScHAL9*+3093Rev-BamHI	CGCTGGATCCTTATTCATAATCC CTAAAAGTGTTCACG
oLC10k90	*ScHAL9*+1Fwd-BamHI	CGCTGGATCCATGGAAAATCAA GGTGGAGATTACAGC
oLC10k91	*ScHAL9*+3093R-PstI	CGCTCTGCAGTTATTCATAATCC CTAAAAGTGTTCACG
oLC10k165	*ScHAL9*_UpIntHygB-392_Fwd	CGAATACCGACTACAGACGG
oLC59	HPH-R	CAGAAACTTCTCGACAGACG
oLC60	HPH-F	CTCGTCCGAGGGCAAAGG
oLC10k166	*ScHAL9*_DnHygB+397_Rev	CGTAAAGCCAATGAATTGGACG
oLC1604	pLC136_GPD_Fwd	TATTGTTTTCTTCACCAACC
oLC9983	*ScHAL9*_orf_133-Rev	GAGTTTCAGGAACCAGACG
oLC883	pLC136_CYC1_Rev	GCGTGAATGTAAGCGTGAC
oLC9982	*ScHAL9*_orf_3010-Fwd	GCTTTTAGAGCCTTATTGCC
oLC386	*CmLEU2*-B	CAGAACCAGAACTCAATGC
oLC387	*CmLEU2*-C	TACAAAATGAACACCACC
oLC10k455	oLC1138+70bp 5UTR *ScMSN4::HygB* pLC3	TCCTTTTTGAATATTTAACGCCTTTATCAGTTCGGCTTTTTTTTCTTTTCTTCTTATTAAAAACAATATAACATGGAGGCCCAGAATACC
oLC10k456	oLC1139+70bp 3UTR *ScMSN4::HygB* pLC3	ATATTATTTCTCCGAAAACTTGTCATACCGTAGCTTGTCTTGCTTTTATTTGCTTTTGACCTTATTTTTTCAGTATAGCGACCAGCATTC
oLC10k457	*ScMSN2::LEU2* 70bp 5UTR pLC43	TTGGTTTTATTTGCTTTATTTTTTCTTTCTTTTTTCAACTTTTATTGCTCATAGAAGAACTAGATCTAAAGCTATGCATCAAGCTTGG
oLC10k458	*ScMSN2::LEU2* 70bp 3UTR pLC43	AAGCTTCATAAGTCATTGAACAGAATTATCTTATGAAGAAAGATCTATCGAATTAAAAAAATGGGGTCTACCTCTAGATGCATGCTCG
oLC10k459	oLC1138+70bp 5UTR *ScHSP12::HygB* pLC3	TATAGAAAAAAAAACCATCTGATTATTCGATAATCTCAAACAAACAACTCAAAACAAAAAAAACTAAATACAACAACATGGAGGCCCAGAATACC
oLC10k460	oLC1139+70bp 3UTR *ScHSP12::HygB* pLC3	CATTGAATATAATATTAAGGAACATCACACATCATAAAGAAAAAACCATGTAACTACAAAGAGTTCCGAAAGACAGTATAGCGACCAGCATTC
oLC10k467	Sc*MSN4*-267bp_Fwd	GCATGAGCATACCTGTAGG
oLC10k468	Sc*MSN4*+118bp_Rev	CCGAATGAAATGACCAACC
oLC10k469	Sc*MSN2*-224bp_Fwd	AACAGACGGAAACCTTGG
oLC10k470	Sc*MSN2*+171bp+Rev	CGTGTATCTAAGTTGTTACAGGC
oLC10k471	*ScHSP12*-220bp_Fwd	CGTTTGGTCAACTTTGAGG
oLC10k472	*ScHSP12*+126bp_Rev	CCACCTCGATTTAAGCGT

oLC10k461	*ScMSN4*-307bp_Fwd	CCTTTCTACCCTCTTGATGC
oLC10k462	*ScMSN4*+294bp_Rev	CGTTAAAACCAGAACGTGC
oLC10k463	*ScMSN2*-313bp_Fwd	GGTAATAGTAAGAAAGCAGCACG
oLC10k464	*ScMSN2*+373bp_Rev	CGATTTTCAAACTGACAGGC
oLC10k465	ScHSP12-312bp_Fwd	GGAAAGGTATACGCAAGC
oLC10k466	ScHSP12+293bp_Rev	GCAACCGCCATGTTTAAGC
oLC10k495	*ScHSP12*-387bp_Fwd	CGTAGTAAAGAGGGGAAAAGG
oLC10k496	*ScHSP12*+372bp_Rev	GGATTTCATGAAAAATCTCGGC
oLC10k497	*ScMSN2*-451bp_Fwd	GTTTCCAGCGAAAGAGACAGG
oLC10k498	*ScMSN2*+569bp_Rev	CATTGAAGCCCCTCTTCATCC
oLC10k499	*ScMSN4*-458bp_Fwd	GAAAACCCGAGCTAGAACTAGG
oLC10k500	*ScMSN4*+461bp_Rev	CCAATCCTTGAATGCTTCCC
oLC805	GPDPro_F_p426GPD	CGGTAGGTATTGATTGTAATTCTG
oLC10k491	*ScHSP12*+1_BamHI Fwd	CGCT**GGATCC**ATGTCTGACGCAGGTAGAAAAGG
oLC10k492	*ScHSP12*+330_SalI Rev	CGCT**GTCGAC**TTACTTCTTGGTTGGGTCTTC
oLC10k493	*ScHSP12*_orf_77-Rev	GCGTATGACTTTTGAGAGTC
oLC10k494	*ScHSP12*_orf_253-Fwd	GCCAAGTCCAAGTTGAACG
oLC10k338	*ScHAL9*_RT_Fwd	GTATTAGCAATCCTGTAGCG
oLC10k339	*ScHAL9*_RT_Rev	CCAATAACCACTCAAGTCTAGC
oLC10k344	*ScACT1*_RT_Fwd	CCTTCTACGTTTCCATCCAAGC
oLC10k345	*ScACT1*_RT_Rev	GCAGTGGTGGAGAAAGAGTAACC
oLC10k346	*ScENA1*_RT_Fwd	GGTGACCCAACAGAAATTGC
oLC10k347	*ScENA1*_RT_Rev	GCTTCACAGTTGAGTCGAATG
oLC10k352	*ScHSP12*_RT_Fwd	GGAATACATCACTGACAAGGC
oLC10k353	*ScHSP12*_RT_Rev	GGCTCCCATGTAATCTCTAGC
oLC10k551	*ScALG9*_RT_Fwd	CACGGATAGTGGCTTTGGTGAACAATTAC
oLC10k552	*ScALG9*_RT_Rev	TATGATTATCTGGCAGCAGGAAAGAACTTGG
oLC10k553	*ScTAF10*_RT_Fwd	ATATTCCAGGATCAGGTCTTCCGTAGC
oLC10k554	*ScTAF10*_RT_Rev	GTAGTCTTCTCATTCTGTTGATGTTGTTGTTG
oLC10k555	*ScUBC6*_RT_Fwd	GATACTTGGAATCCTGGCTGGTCTGTCTC
oLC10k556	*ScUBC*_RT_Rev	AAAGGGTCTTCTGTTTCATCACCTGTATTTGC
oLC5727	*CaurisACT1*+121F	ACCCCAAGTCCAACAGAGAG
oLC5728	*CaurisACT1*+316R	TCCAGCCAAGTCAAGTCTCA
oLC5729	*CaurisGPD1*+141F	ATCCTTGCTGAAAACGCTGC
oLC5730	*CaurisGPD1*+318R	TCCTCGGCCACCTTTACAAT
oLC10k907	*CaurHSP12*_RT_Fwd	GGAGGTTGTTACTGACAAGGTC
oLC10k908	*CaurHSP12*_RT_Rev	CCTCAAGGTACTCCTGAGC

[1] For DMSO solvent triplicates NNNNNN = ATCACG, CGATGT, and TTAGGC, for MMV688766-treated triplicates NNNNNN = CAGATC, ACTTGA, and GATCAG.
